# Phylogeny of a Genomically Diverse Group of *Elymus* (Poaceae) Allopolyploids Reveals Multiple Levels of Reticulation

**DOI:** 10.1371/journal.pone.0078449

**Published:** 2013-11-01

**Authors:** Roberta J. Mason-Gamer

**Affiliations:** Department of Biological Sciences, The University of Illinois at Chicago, Chicago, Illinois, United States of America; J. Craig Venter Institute, United States of America

## Abstract

The grass tribe Triticeae (=Hordeeae) comprises only about 300 species, but it is well known for the economically important crop plants wheat, barley, and rye. The group is also recognized as a fascinating example of evolutionary complexity, with a history shaped by numerous events of auto- and allopolyploidy and apparent introgression involving diploids and polyploids. The genus *Elymus* comprises a heterogeneous collection of allopolyploid genome combinations, all of which include at least one set of homoeologs, designated **St**, derived from *Pseudoroegneria*. The current analysis includes a geographically and genomically diverse collection of 21 tetraploid *Elymus* species, and a single hexaploid species. Diploid and polyploid relationships were estimated using four molecular data sets, including one that combines two regions of the chloroplast genome, and three from unlinked nuclear genes: phosphoenolpyruvate carboxylase, β-amylase, and granule-bound starch synthase I. Four gene trees were generated using maximum likelihood, and the phylogenetic placement of the polyploid sequences reveals extensive reticulation beyond allopolyploidy alone. The trees were interpreted with reference to numerous phenomena known to complicate allopolyploid phylogenies, and introgression was identified as a major factor in their history. The work illustrates the interpretation of complicated phylogenetic results through the sequential consideration of numerous possible explanations, and the results highlight the value of careful inspection of multiple independent molecular phylogenetic estimates, with particular focus on the differences among them.

## Introduction

In the simplest scenario, an allopolyploid genome represents an additive combination of genomes from its contributing diploid species, so the genomic complement of an allopolyploid individual should be identifiable on a gene tree that includes representatives of all of the potential donor species. In reality, however, there are many factors that complicate the phylogenetic characterization of allopolyploids. Some are inherent in the choice of molecular marker. The chloroplast genome typically represents only the maternal parent in angiosperms, and can’t be used to identify the full genomic complement of a polyploid. The highly repetitive internal transcribed spacers (ITS) of the nuclear ribosomal array have the potential to reveal multiple genome donors [1-4], but they are subject to concerted evolution in polyploid genomes [5], so that one or more of the progenitors’ arrays might be unrepresented on an ITS gene tree. 

These problems can be largely avoided with the use of low-copy nuclear genes; they are biparentally inherited and are less likely to undergo gene conversion, so their gene trees have the potential to reveal all of an allopolyploid’s genome donors. As a result, phylogenetic analyses of low copy nuclear genes have been fruitful for clarifying the origins of a wide assortment of allopolyploid species [6-24]. However, there are numerous phenomena that can confound the identification of allopolyploid individuals in phylogenetic analyses of low-copy genes. For example, polyploidization can be associated with extensive genome reorganization, including homoeolog loss (e.g., [18,25-29]); as a result, one or more homoeologs can be missing from a given gene tree, and an allopolyploid’s origins can be obscured. The effect will vary across gene trees depending on whether the maternal, paternal, or neither copy is lost. Single-gene duplication, leading to two or more paralogous copies, can also cause misleading phylogenetic estimates. Paralogy is potentially detectable, and is even quite informative in its own right if gene copies are thoroughly sampled [20,30-37]. However, unsuspected paralogy coupled with limited within-individual sampling and/or paralog loss can result in a confusing gene tree on which each allopolyploid individual is represented by a random selection of one or more paralogs. The identification of a polyploid’s progenitors can be further confounded if the relevant diploids are missing from the analysis. In these cases, a polyploid will exhibit gene copies with no clear origin among the diploids. The causes for missing diploids include sparse sampling, and the more intractable problem where one or more of a polyploid’s donors are undiscovered or extinct (e.g., [8,20,23,38-41]).

Speciation following polyploidization will yield monophyletic clades of polyploids nested within the donor groups, but subsequent hybridization among them [22,42] will lead to incongruence within those clades across trees. Polyploid origins can be even more difficult to unravel when allopolyploid species form recurrently (e.g., [10,18,43-45]). Subsequent hybridization among them [19,20,22,46-50] can yield individuals with alleles indirectly acquired from many different diploid individuals. Introgression among diploid species prior to polyploidization [40,47,49,51,52] can lead to similar outcomes, in which an allopolyploid has obtained gene copies representing multiple diploid donors. Finally, although a difference in ploidy level is often viewed as an effective barrier to gene exchange, there have been numerous reports of introgression from diploids to polyploids [48,49,53-56], which can further enrich a polyploid’s genome while obscuring its phylogenetic history. All of these scenarios - introgression among polyploids, among diploids prior to polyploidization, and among ploidy levels – can lead to conflicting placement of a polyploid’s homoeologs on one or more gene trees; thus, conflict can be difficult to interpret in terms of specific phenomena [8,57]. 

This study focuses on *Elymus*, a diverse, polyphyletic assemblage of allopolyploid species in the wheat tribe, Triticeae Dumort. (A recently-discovered use of the name Hordeeae in 1820 – I. I. Martinov, *Tekhno-Bot. Slovar*.: 314, Aug 1820 – has priority over Triticeae – B. C. J. Dumortier, *Observ*. *Gramin*. *Belg*.: 82, Jul–Sep 1824. I use the prevailing name Triticeae here, pending the outcome of a planned proposal to retain its use [M. E. Barkworth, pers. comm.].) While wheat is the most familiar allopolyploid in the tribe (and perhaps among plants), polyploidy has impacted the history of the entire group – about 75% of the approximately 300 species are of polyploid origin [58]. Cytogenetic analyses reveal that the genus *Pseudoroegneria* (genome designation **St**) is a prolific contributor to the tribe’s allopolyploids. The **St** genome is found in combination with genomes from numerous other genera, including *Hordeum* (genome **H**), *Australopyrum* (**W**), *Agropyron* (**P**), and an unknown donor with genome designation **Y**. This heterogeneous group of allopolyploid combinations is often collectively classified as *Elymus* sensu lato, as reflected in numerous major floristic works [59-62]. Under this definition, *Elymus* comprises **StStHH** and **StStYY** tetraploids, and **StStStStHH**, **StStHHHH**, **StStStStYY**, **StStYYYY**, **StStHHYY**, **StStYYWW**, and **StStYYPP** hexaploids [58,63-78]. This group, while united by their shared **St** genome, is obviously polyphyletic, and there has been recent work toward updating their classification to better reflect differences in genomic content [79-82]. There are some additional, higher-order polyploids that include the **St** genome but are not classified as *Elymus* s.l. For example, *Pascopyrum smithii* (Rydb.) Á.Löve combines the **St** and **H** genomes with the **Ns** genome of *Psathyrostachys* in an **StStHHNsNsNsNs** configuration [83]. The **St** genome also co-occurs with the **E** and/or **J** genomes of *Thinopyrum* [84-86] in complex hexa- and decaploid configurations that might also include full or partial *Taeniatherum*, *Crithopsis*, *Aegilops*, and/or *Dasypyrum* genomes [87,88].

**Figure 1:**
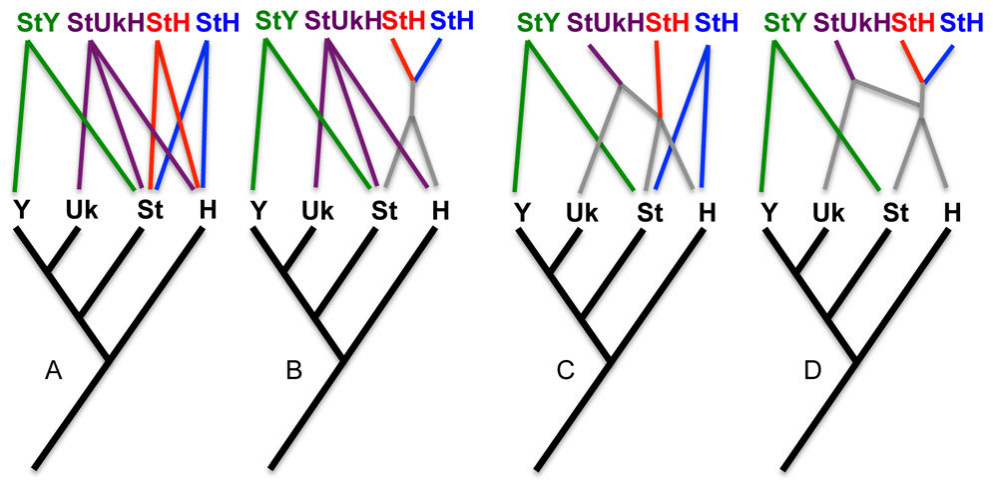
Evolutionary scenarios. Four scenarios representing separate (a) or partially sequential (b–d) origins of the four provisional categories of polyploids: **StStHH** tetraploids native to North American (blue) and Eurasia (red), Asian **StStYY** tetraploids (green), and *E. repens* hexaploids (purple). Additional scenarios are conceivable, including recurrent origins of each group.

The present work is focused primarily on tetraploid species along with one allohexaploid. As a starting framework, the included species are provisionally divided into four groups (Figure 1). The first two groups include the Eurasian and North American **StStHH** tetraploids, respectively. Their allopolyploid origins from *Hordeum* and *Pseudoroegneria* were initially revealed through cytogenetic analyses (e.g., [66,89-92]) and confirmed in molecular phylogenetic analyses [93-97]. The third group includes **StStYY** tetraploids (=*Roegneria* as used by Yen et al. [81]) which comprise 30–40 species distributed throughout central Asia [73]. Cytogenetic [66,68,71-74,76-78,98-101] and molecular phylogenetic analyses [40,102,103] clearly show the allotetraploid nature of these species, and reveal *Pseudoroegneria* as a genome donor (**St**), but they have been largely inconclusive about the identity of the **Y**-genome donor. The fourth group includes six individuals of *Elymus repens* L. Gould, the sole hexaploid species (2n=42) included in the study. The species is native to Europe and Asia, but has become a widespread weed throughout much of the United States and eastern Canada [104]. Cytogenetic analyses of its genome content based on chromosome pairing [105-110] and genomic in situ hybridization [111] are equivocal individually, but taken together, they suggest that *E. repens* has two similar genome sets derived from *Pseudoroegneria* and a third set from *Hordeum* (**StStStStHH**). Molecular phylogenetic analyses provide a more complex picture, suggesting three distinct donors, and introgression from at least one additional genus within the tribe [52,112,113], along with apparent contributions from tribes Bromeae and Paniceae [52], and Poeae [113].

The four provisional polyploid groups have been examined individually, and here the relationships among them are investigated using data from two chloroplast DNA regions, and three single-copy nuclear genes. The specific goals are to determine: (1) whether the four provisional groups represent phylogenetically distinct lineages; and (2) whether specific evolutionary phenomena (homoeolog loss, paralogy, progenitor extinction, multiple polyploid origins, and/or introgression) can be identified, or excluded, as explanations for reticulate patterns beyond allopolyploidy itself.

## Materials and Methods

### Data sets

The samples include allopolyploid representatives of *Elymus* from the four provisional groups, along with a broad sample of monogenomic, mostly diploid representatives from throughout the tribe (Table S1). The chloroplast DNA (cpDNA) data set (Dataset S1) includes the trnT-trnL-trnF region, and the rpoA gene. Sequences are derived from several previous analyses (see Table S1 footnotes) with the addition of new sequences representing 16 Eurasian **StStHH** and **StStYY** tetraploid *Elymus* accessions and eight diploid *Pseudoroegneria* accessions (Table S1). The trnT-trnL-trnF sequences were amplified using *a* and *f* primers, and sequenced with *a*, *b*, *c*, *d*, *e*, and *f* primers [114]. The rpoA gene was amplified using rpoA1 and rpoA2 primers, and sequenced with rpoA1, rpoA2, rpoA4, rpoA5, rpoA8, and rpoA9 primers [115]. Amplification and sequencing followed methods previous applied to this tribe [116]. 

 Each of the nuclear gene data sets – phosphoenolpyruvate carboxylase (pepC; Dataset S2), β-amylase (Dataset S3), and granule-bound starch synthase I (GBSSI; Dataset S4) – combine data from numerous prior analyses (see Table S1 footnotes) that were primarily designed to reveal the relationships of the individual polyploid groups to their diploid progenitors. The present analyses were undertaken to further clarify the relationships among these diverse groups. In addition, a phylogenetically broader analysis of GBSSI exon data includes additional representatives of the Pooideae, and a few representatives of the Bambusoideae (Table S2; Dataset S5); this analysis was used to clarify the placement of a divergent clade of sequences from the *E. repens* hexaploids. 

### Phylogenetic analyses

Three of the data sets were partitioned for model specification (Table S3). The chloroplast DNA data were partitioned by region: the trnT-trnL-trnF region, which consists primarily of non-coding sequences of the intergenic spacers and the trnL intron, and the rpoA gene, which consists mainly of protein-coding sequence. The β-amylase and starch synthase gene data sets were both partitioned into exons vs. introns. The phosphoenolpyruvate carboxylase sequences were analyzed under a single model, because over 90% of the data set represents a single intron, with only small portions of exons 1 and 2 (59 and 23 bp, respectively). The GBSSI exon data used in the broader phylogenetic analysis were also analyzed under a single model.

For each partition, jModelTest [117] was used to select among 24 candidate models. These include three substitution models (AC=AG=AT=CG=CT=GT; AC=AT=CG=GT ≠AG=CT; and AC≠AG≠AT≠CG≠CT≠GT), first under an assumption of equal nucleotide frequencies: JC [118], K80 [119], and SYM [120] – and then allowing for unequal nucleotide frequencies: F81 [121], HKY [122], and GTR [123]. Each of these was combined with four among-site rate parameters: equal rates, some proportion of sites invariant (I)[122], gamma-distributed rate variation (Γ)[124], or I+Γ [125,126]. Input files were formatted for jModelTest using ALTER [127]; model parameters were estimated on maximum-likelihood (ML) estimated trees and models were selected using the Akaike information criterion [128]. 

**Figure 2:**
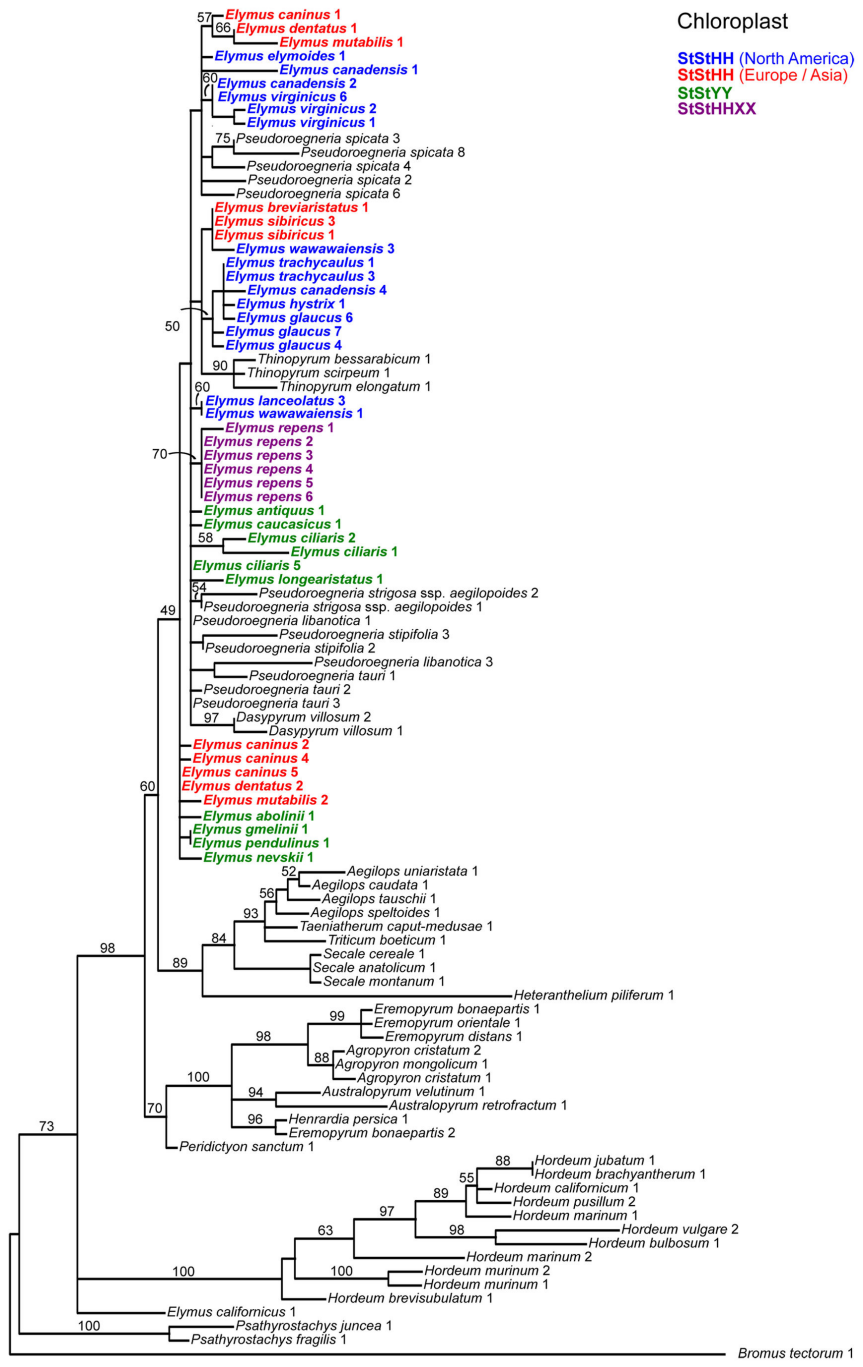
Chloroplast DNA gene tree. The data set (Dataset S1) was partitioned by genome region for *a* *priori* model specification in jModelTest, and simultaneous parameter estimation in GARLI: the trnT-trnL-trnF region (GTR+Γ) and the rpoA gene (GTR+Γ). This represents the best tree from 50 GARLI search replicates. ML bootstrap results are based 100 GARLI replicates under the same models as used in the tree searches. Monogenomic representatives of the tribe are in black font; *Elymus* representatives are in colored, boldface font. Colors distinguish the four hypothetical *Elymus* species groups as described in the text: North American (blue) and Eurasian (red) **StStHH** tetraploids, Asian **StStYY** tetraploids (green), and *E. repens* hexaploids (purple).

**Figure 3:**
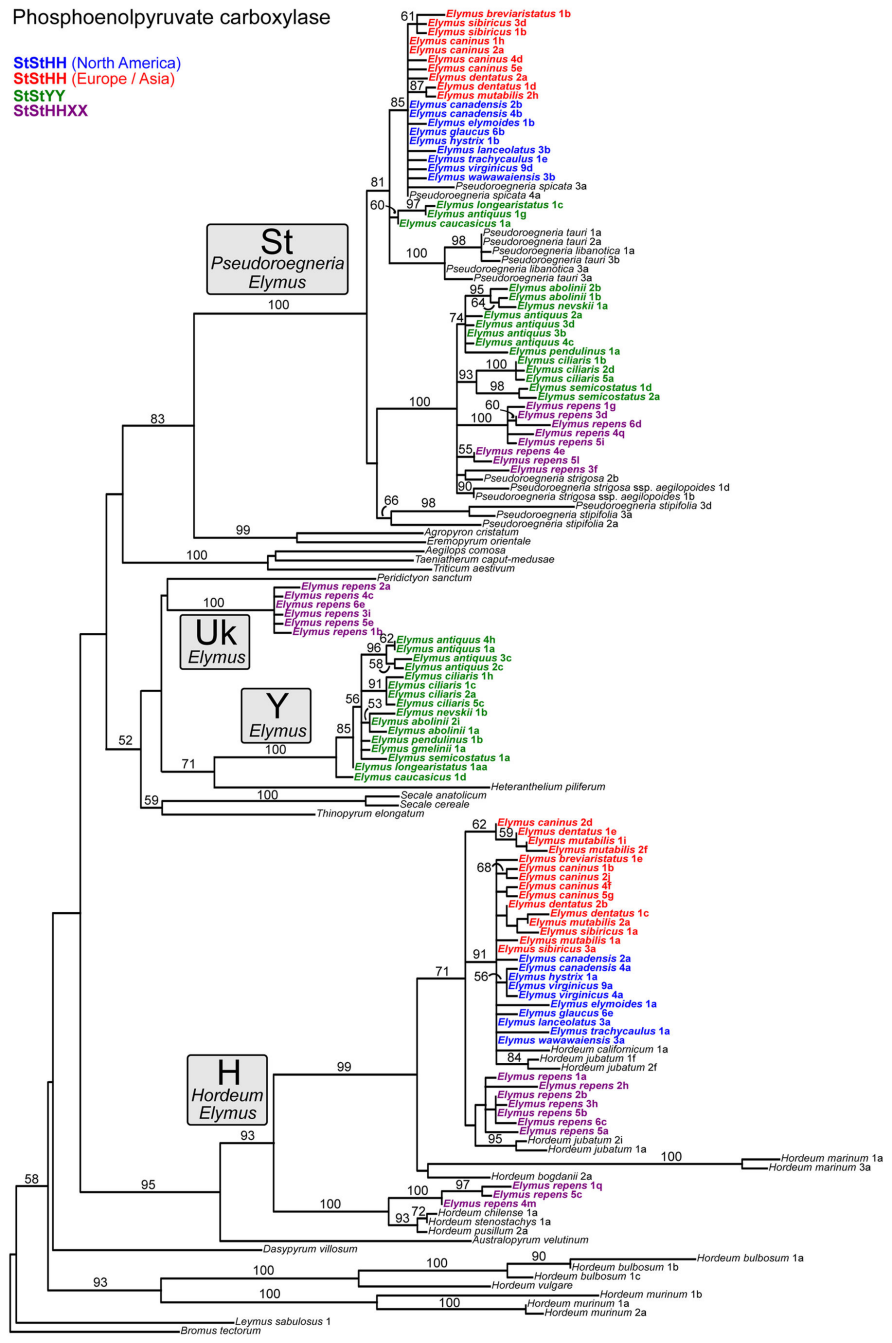
Phosphoenolpyruvate carboxylase gene tree. The data set (Dataset S2) was treated as a single partition for *a* *priori* model specification in jModelTest (HKY+Γ), and simultaneous parameter estimation in GARLI. This represents the best tree from 50 GARLI search replicates. ML bootstrap results are based 100 GARLI replicates under the same models as used in the tree searches. Font colors follow Figure 2.

**Figure 4:**
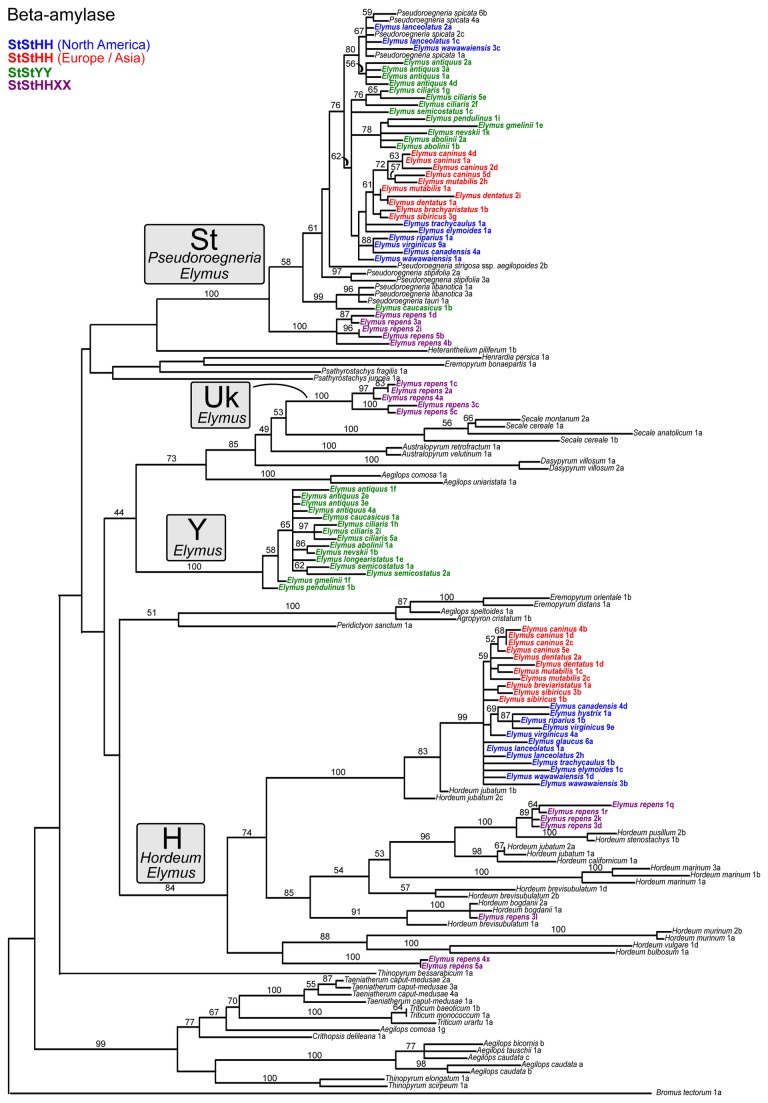
Beta amylase gene tree. The data set (Dataset S3) was partitioned for *a* *priori* model specification in jModelTest, and simultaneous parameter estimation in GARLI: exons (K80+Γ) and introns (GTR+I+Γ). This represents the best tree from 50 GARLI search replicates. ML bootstrap results are based 100 GARLI replicates under the same models as used in the tree searches. Font colors follow Figure 2.

**Figure 5:**
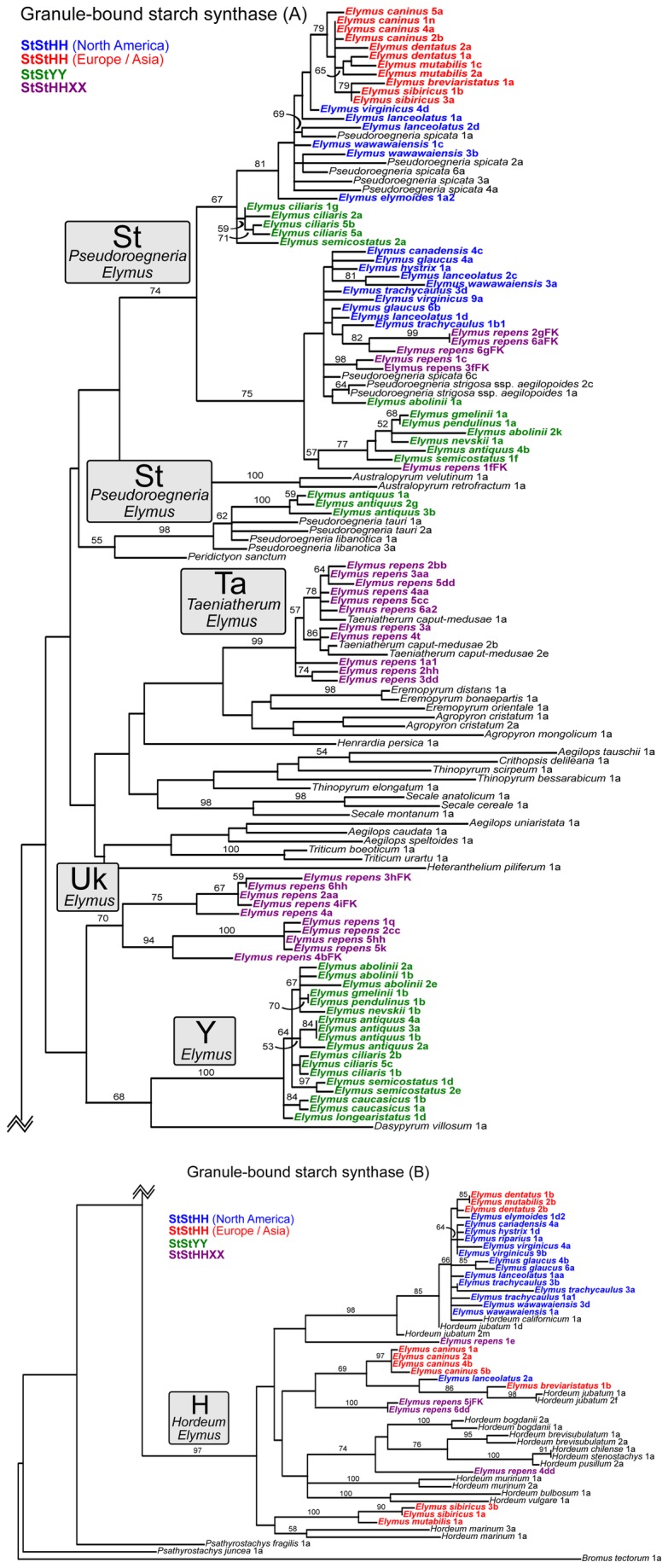
Granule-bound starch synthase I gene tree. The data set (Dataset S4) was partitioned for *a* *priori* model specification in jModelTest, and simultaneous parameter estimation in GARLI: exons (GTR+Γ) and introns (GTR+Γ). This represents the best tree from 50 GARLI search replicates. ML bootstrap results are based 100 GARLI replicates under the same models as used in the tree searches. Font colors follow Figure 2.

**Figure 6:**
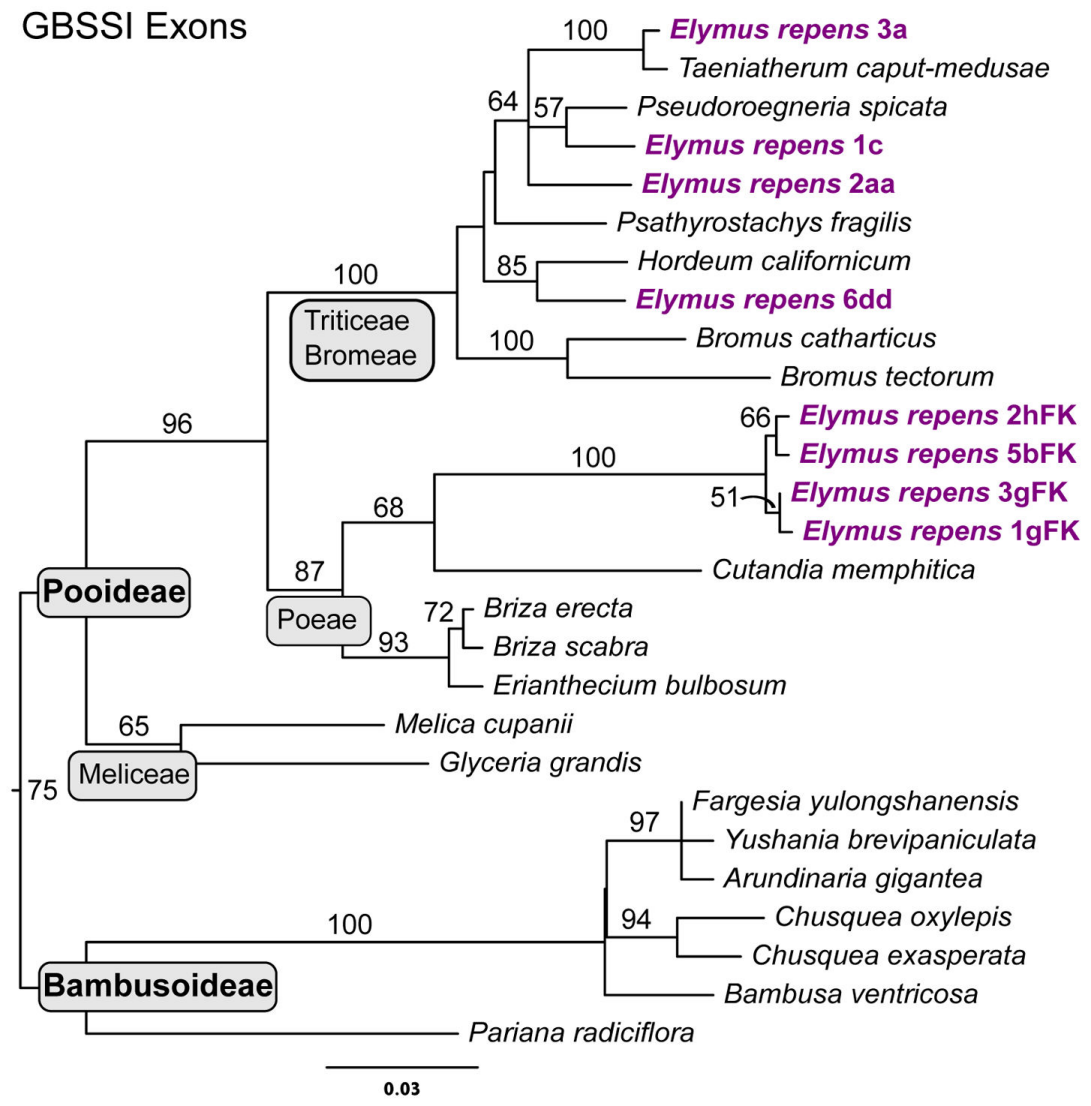
Granule-bound starch synthase I exon tree: Pooids and Bambusoids. The data set (Dataset S5) was treated as a single partition for *a* *priori* model specification in jModelTest (GTR+I+Γ), and simultaneous parameter estimation in GARLI. ML bootstrap results are based 100 GARLI replicates under the same model. The Triticeae clade represents a small subset of the individuals in the full GBSSI tree (Figure 5). *Elymus repens* sequences are highlighted in purple, boldface font.

Maximum-likelihood phylogenetic analyses were run under the selected models using GARLI [129] v. 2.0 (http://garli.googlecode.com). Fifty analyses were run for each data set with random starting topologies, for an unlimited number of generations, and automatic termination following 10,000 generations without a meaningful change in score (ln L increase of 0.01). The tree with the best score was chosen to depict each gene tree (Figures 2–6), and for final model parameter estimates (Table S3). Bootstrap support estimates were based on 100 ML replicates under the same model and under the same conditions, except that the change requirement for search termination was increased from 0.01 to 0.02.

## Results

### Chloroplast DNA tree

 The cpDNA data (Figure 2) place the sequences from all of the polyploid *Elymus* individuals into a single weakly-supported clade, along with *Dasypyrum*, *Pseudoroegneria*, and *Thinopyrum*. Thus, all species of this fairly diverse, polyphyletic sample of *Elymus* species appear to share the same maternal donor. The consistent placement of *Elymus* with *Pseudoroegneria* on other gene trees (below), and never with *Dasypyrum* or *Thinopyrum*, points to *Pseudoroegneria* as the maternal donor to *Elymus*. *Elymus* terminal branch lengths are short and there is very little resolution among the individuals. The only well-supported groups within the large clade are those that group the two *Dasypyrum villosum* individuals (97%) and the three *Thinopyrum* individuals (90%); the six *E. repens* sequences are grouped with moderate (70%) support. None of the three pre-defined tetraploid groups (Eurasian **StStHH**, North American **StStHH**, or Asian **StStYY**) form clades. The separation of the sole North American *Pseudoroegneria* species (*P. spicata*) from the Eurasian species on the ML tree lacks meaningful support. Thus, although the addition of 24 species of *Elymus* and *Pseudoroegneria* provides a much broader geographic sample than previous analyses, the tree reveals little about the role of geography, or the independent formation of multiple polyploid combinations, in the patterns of cpDNA differentiation among the species. 

### Phosphoenolpyruvate carboxylase

 Within the **St** clade on the pepC tree (Figure 3), the sequences from both North American and Eurasian **StStHH** *Elymus* are grouped with *P. spicata* (85%), the only native North American *Pseudoroegneria* species. There is little structure within this clade, and the sequences are very similar to one another. Sequences from *E. repens* and the tetraploid **StStYY** Asian species are grouped with *P. strigosa* (100%), a Eurasian species. The only exception to this involves three **StStYY** species: *E. antiquus* (one out of four individuals), *E. caucasicus*, and *E. longearistatus*; their **St** sequences are closer to (though not within) the “wrong” clade in terms of phylogenetic relationship and genetic similarity. The other three *E. antiquus* individuals are in the main group of **StStYY** species.

 The **H**-sequences from the North American and Eurasian **StStHH** species are closely related to one another (as in the **St** clade), and to those from *E. repens*. They group with diploid *H. californicum* and tetraploid *H. jubatum* (71%), which are both North American natives. There is little phylogenetic structure among these sequences, but most of the sequences from the *Elymus* tetraploids are closely related to *H. californicum* and one genome from *H. jubatum*, while *E. repens* is weakly grouped with the other genome of *H. jubatum*. Three sequences, representing *E. repens* individuals 1, 4, and 5, form a separate well-supported clade (100%) outside of the main *Elymus* clade, with *H. chilense* and *H. stenostachys* (both South American), and *H. pusillum* (North American). Two of these individuals, 1 and 5, also have copies in the main clade.

 The **Y**-sequences from the **StStYY** individuals form a phylogenetically distinct clade (100%) with a moderately-supported relationship (71%) with *Heteranthelium piliferum*. *Elymus antiquus*, which was polyphyletic in the **St** clade, is strongly monophyletic (96%) in the **Y** clade. An additional clade, representing a third clade of *E. repens* sequences of unknown origin (Figure 3, “UK”), is phylogenetically distinct from the **St**, **H**, and **Y** sequences. The origin of this clade is unclear; its association with the **Y**-sequence clade and *H. piliferum* is very weak (<50%). The “UK” and **Y** sequences are generally in the same part of the tree, but support for a close relationship between them, and thus any suggestion that the “UK” and **Y** sequences are from the same unknown genome donor, is weak at best.

### Beta-amylase

On the β-amylase tree (Figure 4), the **St** sequences from the **StStHH** and **StStYY** tetraploids form a clade with North American *P. spicata* (76%). The single exception is one *E. caucasicus* sequence with a strong (99%) relationship to three Iranian *Pseudoroegneria* accessions – two of *P. libanotica* and one of *P. tauri*. The **StStHH** *Elymus* / *P. spicata* relationship reflects the results on the pepC tree (Figure 3), but the inclusion of the sequences from the **StStYY** sequences is in sharp contrast. The sequences from Eurasian **StStHH** species form a weak monophyletic subgroup (61%), while those from North American **StStHH** species and from the **StStYY** species are polyphyletic. The *E. repens* **St** sequences form a phylogenetically distinct monophyletic group (100%), sister to the remaining **St** sequences. 

The **H** sequences of all of the **StStHH** species form a strong clade (99%) that is closely related (100%) to one of the genomes of the tetraploid North American species *H. jubatum* (sequences 1b and 2c). As on the pepC tree, some of the sequences from *E. repens* form a close relationship (100%) with those from *H. stenostachys* and *H. pusillum*. These in turn group with *H. californicum* and the other *H. jubatum* homoeologs (sequences 1a and 2a; 96%). In sharp contrast to the pepC tree, none of the *E. repens* **H** sequences group with those from **StStHH** tetraploids. One of them (3l) groups (91%) with two accessions of *H. bogdanii* representing Tajikistan and China, and two (4x and 5a) are together (100%) on a long branch that is not closely associated with any of the sampled *Hordeum* species. 

The **Y**-genome sequences from the **StStYY** tetraploids again form a phylogenetically distinct clade (100%). In contrast to the pepC tree, this tree shows no evidence of a relationship between the **Y**-genome clade and *H. piliferum*; furthermore, they show no clear association with any of the diploid donors. The *E. repens* “UK” sequences again form a monophyletic group (100%), this time within a larger, moderately-supported (85%) group with *Australopyrum*, *Dasypyrum*, and *Secale*. As on the pepC tree, the “UK” clade shows no apparent close relationship to the **Y**-genome clade of the **StStYY** tetraploids. 

### Granule-bound starch synthase

The **St**-genome sequences from *Elymus* show close relationships to three different *Pseudoroegneria* species (Figure 5a). First, the **St** sequences from Eurasian **StStHH** tetraploids, and about half of those from the North American tetraploids, form a monophyletic group (81%) with North American *P. spicata*. Sequences from two of the **StStYY** species, *E. ciliaris* and *E. semicostatus*, are weakly (67%) linked to this group as well. Second, the remaining sequences from the North American **StStHH** tetraploids, most from the **StStYY** species, and all from hexaploid *E. repens* form a clade with *P. strigosa* and a single *P. spicata* sequence (75%). Within this clade, none of the species groups (**StStHH**, **StStYY**, *E. repens*) are monophyletic. Finally, the **St** sequences from three of the four **StStYY** *E. antiquus* individuals are strongly (98%) grouped with *P. tauri* and *P. libanotica* (four accessions, all representing Iran). These sequences are, at best, only weakly associated with the main St group.

The relationships among the **H**-genome sequences from *Elymus* and *Hordeum* (Figure 5b) are complicated. On one hand, the results partly reflect those on the pepC and/or β-amylase trees: most of the sequences from the North American **StStHH** tetraploids, and several from the Eurasian **StStHH** tetraploids, are closely related to the North American *Hordeum* species *H. jubatum* (allotetraploid) and *H. californicum* (diploid). On the other hand, the remaining **H**-genome sequences (including one from a North American tetraploid, most from the Eurasian tetraploids, and all from hexaploid *E. repens*) are scattered among the other *Hordeum* species. Some of the tetraploids’ sequences are grouped with the other genome of the allotetraploid *H. jubatum*, suggesting possible involvement between *Elymus* and *Hordeum* at the tetraploid level. The three remaining sequences from the Eurasian tetraploids (*E. sibiricus* 1a and 3b, and *E. mutabilis* 1a) and the *E. repens* sequences are on long branches with no clear relationship to any particular *Hordeum* species.

The **Y**-genome sequences of the **StStYY** tetraploids (Figure 5a) again form a well-supported clade (100%); this tree uniquely suggests a weak relationship (68%) to *Dasypyrum villosum*. The *E. repens* “UK” sequences form a moderately supported group (70%). As on the pepC tree, the *E. repens* “UK” sequences are very weakly associated with the **Y** clade, but again, there is no clear suggestion that they are derived from the same unknown diploid donor. 

 The GBSSI tree shows a unique, fourth group of *E. repens* sequences very closely associated (99%) with *Taeniatherum* (**Ta**; Figure 5a). All of the *E. repens* individuals are represented in this clade, and no related sequence is found in any of the tetraploids. Thus, the *E. repens* genome harbors four distinct GBSSI copies within the Triticeae. In addition, three *E. repens* individuals have a divergent fifth GBSSI copy type that falls outside of the tribe (Figure 6). The sample of non-Triticeae pooid taxa is limited, but this divergent clade of *E. repens* sequences clearly fall outside of the well-supported Triticeae /Bromeae clade, and apparently within the Poeae. 

## Discussion

### General relationships among lineages

The provisional division of *Elymus* into four groups (North American **StStHH** tetraploids, Eurasian **StStHH** tetraploids, Asian **StStYY** tetraploids, and the Eurasian hexaploid *E. repens*) reflects a simple preliminary hypothesis that the groups represent distinct evolutionary lineages, whether arising independently from one another (Figure 1a) or partially sequentially (Figure 1b–d). The placement of *Elymus* individuals on each the four gene trees can highlight deviations from the hypothesis that all – or any – of the groups represent straightforward lineages. In summary, the data support three independently-derived lineages (Figure 1b): a combined North American and Eurasian **StStHH** lineage, the **StStYY** species, and *E. repens*. All three, however, show complicated phylogenetic patterns, as discussed below under the heading “Potentially confounding phenomena.”

### The cpDNA tree does not distinguish lineages

Of the four trees, the cpDNA tree (Figure 2) is the least informative with respect to the origins and evolution of the polyploid lineages, except in its suggestion that *E. repens* was derived from one (or multiple similar) maternal donor(s). This is due largely to lack of resolution within the large *Pseudoroegneria* / *Elymus* clade. All three tetraploid groups are polyphyletic on this tree, but with support too weak to convincingly suggest actual polyphyletic origins. This tree, when considered alone, suggests three potential chloroplast donors: *Dasypyrum*, *Pseudoroegneria*, or *Thinopyrum*, but arguments in favor of *Pseudoroegneria* as the maternal have already been presented in detail elsewhere [116]. Briefly (1), there are no cytogenetic or molecular phylogenetic data that point to either *Dasypyrum* or *Thinopyrum* as a contributor to the *Elymus* polyploids; and (2) a *Dasypyrum* / *Thinopyrum* / *Pseudoroegneria* relationship appears on no other phylogenetic estimates to date, suggesting that *Dasypyrum* and *Thinopyrum* are “misplaced” with *Pseudoroegneria* on the cpDNA tree as a result of past introgression. 

### The North American and Eurasian StStHH tetraploids are united, but with signs of introgression

**Figure 7:**
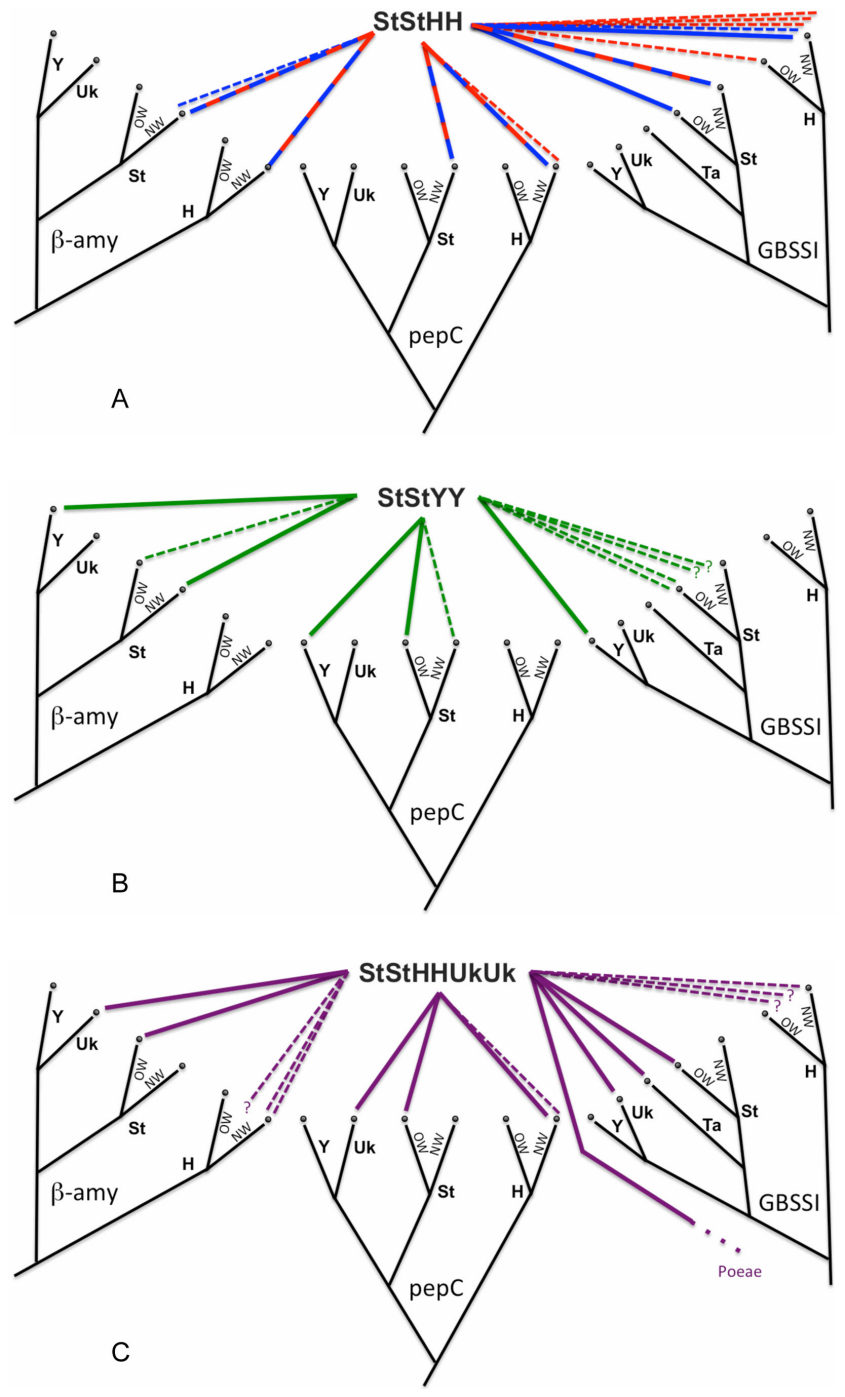
Summary of *Elymus* genetic diversity. The black trees represent diploid phylogenies; basal relationships are poorly supported on the actual gene trees, so some relationships among **St**, Y, H, and “UK” are unclear. The colored lines represent contributions to polyploids; unbroken and dotted lines represent major and minor contributions, respectively. Colors follow Figures 1–5. The **St** (*Pseudoroegneria*) and **H** (*Hordeum*) species are distinguished as Old World (OW) and New World (NW). a. **StStHH** species. The red-and-blue lines indicate where the North American (blue) and Eurasian (red) groups received major contributions from the same, or phylogenetically similar, donors. The β-amylase and pepC trees suggest fairly straightforward New World origins, with few minor St- or H-genome contributions. The GBSSI tree shows more complicated origins, especially with respect to *Hordeum*’s contribution. b. **StStYY** species. Without any representative Y-genome diploids, a single donor is hypothesized based on monophyly and sequence similarity (Figures 3–5). Primary St-genome donors are a mix of New World (β-amylase) and Old World (pepC and GBSSI), with minor contributions from the alternate region in all three cases. c. *Elymus repens*. All trees are at least consistent with a single Old World St-genome contribution. The presence of a third (“UK”) clade on all trees reveals an unknown genome donor. There is no single, major H-genome contributor, but a mix of multiple contributors. Contributions unique to the GBSSI tree (from *Taeniatherum*, and from an unknown species from the tribe Poeae) are consistent with introgression after polyploidization.

Some previous studies suggested separate origins of the Eurasian and North American **StStHH** species groups from Eurasian and North American progenitors, respectively [97,130,131]. In contrast, the results here point to a North American origin for both groups. The three nuclear genes, considered individually and in combination, support a close relationship between the Eurasian and North American **StStHH** tetraploid groups, but with evidence of introgression (Figure 7a). The phosphoenolpyruvate carboxylase and β-amylase trees generally unite the **StStHH** tetraploids and are consistent with a North American origin; their **St** genomes are closely related to the North American native diploid *P. spicata*, and their **H** genomes are closely related to the North American species *H. californicum* and tetraploid *H. jubatum* (which itself has a *H. californicum*-like donor [38]). The GBSSI tree is more difficult to interpret, with both the North American *Elymus* **St** sequences, and the Eurasian *Elymus* **H** sequences, polyphyletic. This pattern is seen on only one gene tree, so it is more likely an indicator of GBSSI introgression than of multiple origins.

### The Asian *StStYY* tetraploids probably represent a single evolutionary lineage with subsequent introgression

The **Y**-genome of the Asian tetraploids represents a phylogenetically distinct entity. It does not fit into an existing genus, as shown here and in earlier studies [132,133] (although one previously-published gene tree shows a close relationship between **Y**-genome sequences and *Australopyrum* [103]). The hypothesis that the **Y** genome is a derivative of the **St** genome [134,135] is not supported here. Thus, the proposed application of separate generic status (*Roegneria* [81]) to the cytogenetically distinct **StStYY** is further justified by phylogenetic data. The donor of its **St** genome is unequivocally *Pseudoroegneria*, but the nuclear gene trees are in conflict with regard to which species is the most likely donor. While the pepC tree points to the Eurasian species *P. strigosa* as a likely donor, the β-amylase tree instead indicates *P. spicata*, placing the **StStHH** species in a largely North American clade with the **StStHH** species. The sequences are widely polyphyletic on the GBSSI tree, again suggesting a history of introgression (Figure 7b).

***Elymus repens* represents a single, very complicated lineage**. In spite of its highly polyphyletic **H**-genome sequences on all three nuclear trees (Figures 3–5, 7c), *E. repens* as sampled here probably represents a single lineage. This is supported by numerous shared genetic characteristics, including a third “unknown” genome (Figures 3–5) and a *Taeniatherum*-like GBSSI allele (Figure 5), features that are also shared by individuals sampled from their native range [52]. Based on its presumed **StStStStHH** genomic content, the hexaploid *E. repens* might conceivably be derived from an existing tetraploid **StStHH** lineage, and with the uncertainties about its donors, it is even difficult to rule out an **StStYY** progenitor a priori. The phylogenetic data are inconsistent. For example, the pepC **H**-genome sequences (Figure 3) clearly link *E. repens* with the **StStHH** tetraploids and North American *Hordeum* species, while the **St** sequences on the same tree group *E. repens* strongly with the **StStYY** species and a Eurasian *Pseudoroegneria* species, *P. strigosa*. The β-amylase **St** and **H** sequence data (Figure 4) separate *E. repens* from both the **StStHH** and the **StStYY** tetraploids, while the GBSSI **St** sequences places it with individuals from both groups. Without a consistent relationship between *E. repens* and the **StStHH** or **StStYY** tetraploids, I tentatively conclude that the origin of *E. repens* is independent of the sampled tetraploids. However, with extensive conflict among data sets, the question is not fully resolved.

### Potentially confounding phenomena

 In phylogenetic analyses of allopolyploids and their diploid relatives, non-conflicting relationships across gene trees suggest an uncomplicated evolutionary history, while discordance among trees suggests a history of reticulation beyond allopolyploidization itself, such as introgression among polyploid lineages and/or their progenitors. As outlined in the Introduction, numerous evolutionary phenomena (Table 1) can affect the placement of polyploids on gene trees. These are discussed below with respect to the present results from the wheat tribe analyses, with the caveat that they can be difficult to pin down individually, not only because some have similar outcomes, but also because they can act simultaneously and obscure one another’s effects. 

**Table 1:** Phenomena that affect polyploid phylogenies.

**Phenomenon**	**Similar effect on all trees?**	**Affects diploids or polyploids?**	**Potential effects**	**Proposed examples**
1. Homoeolog loss following polyploidization	No	Polyploids	Placement of polyploid varies depending on which copy is retained.	*Tragopogon* [25]; *Solanum* [18]; *Triticum* [26]; Review [29]; *Brassica* [27]; *Brassica*, *Arabidopsis* [28].
2. Paralogy; paralogs retained and intensively sampled	No	Both	Intra-individual polymorphism shared by all species with duplication; duplicate clades on tree.	*Gossypium* [33]; *Viola* [35]; *Paeonia* [32,36]; Brassicaceae, Cleomaceae [34]; Rosaceae [30]; Gesneriaceae [31]; *Viburnum* [37].
3. Paralogy; limited paralog sampling	No	Both	Sporadic intra-individual polymorphism, polyphyletic taxa, incongruence with other gene trees.	–
4. Paralogy; loss of paralogs	No	Both	Sporadic intra-individual polymorphism, polyphyletic taxa, incongruence with other gene trees.	*Mentzelia* [20]; *Glycine* [16].
5. Diploid progenitor(s) extinct, undiscovered, or unsampled	Yes	Polyploids	One or more homoeologs unassociated with any diploid.	*Hordeum* [38]; *Mentzelia* [20]; *Cerastium* [8]; *Cardamine* [11]; *Elymus* [40]; *Microserus* [41]; *Dryopteris* [23].
6. Recurrent origins of a polyploid combination	Yes	Polyploids	Polyploid sequences polyphyletic within donor clades and congruent among gene trees.	*Spiranthes* [43]; *Solanum* [18]; *Tragopogon* [44]; *Rosa* [10]; Review [45].
7. Introgression among genomically similar recurrent polyploids	No	Polyploids	Polyploid sequences polyphyletic within donor clades and incongruent among gene trees.	*Centauria* [47]; *Dactylorhiza* [48]; *Viola* [22].
8. Introgression among species derived from a single polyploid ancestor	No	Polyploids	Polyploid sequences monophyletic within donor clades and otherwise incongruent among gene trees.	*Spartina* [42]; *Viola* [22].
9. Introgression among genomically distinct polyploid lineages	No	Polyploids	Polyploid sequences broadly polyphyletic across multiple donor clades and incongruent among gene trees.	*Galeopsis* [19]; *Mentzelia* [20]; *Houstonia* [46]; *Arabidopsis* [49]; *Elymus* [50].
10. Introgression from diploid to polyploid	No	Polyploids	Polyploid sequences polyphyletic within donor clades and incongruent among gene trees.	*Senecio* [53]; *Dactylorhiza* [48]; *Arabidopsis* [49]; *Achillea* [54]; *Dodecatheon* [55]; *Betula* [56].
11. Introgression among diploids prior to polyploidization	No	Both	Sequences from diploids and their derived polyploids are polyphyletic and incongruent among gene trees.	*Glycine* [51]; *Centauria* [47]; *Arabidopsis* [49]; *Elymus* [52]; *Elymus* [40].

Multiple homologous gene copies (Table 1, 1–4) can mislead phylogenetic analyses if they are unsuspected, incompletely sampled, or have undergone copy losses. These include homoeologs (the homologous gene copies representing different genome sets in a polyploid), and paralogs (used here to refer only to copies arising through within-genome gene duplication). Homoeolog loss following polyploidization (Table 1, 1) has occurred rarely, if at all, for the genes examined. Occasional homoeologs are missing from some taxa (NR in Table S1), but at least for these three genes, copy loss is clearly not widespread in *Elymus*. The occasional missing copies might represent individual instances of copy loss, failure to amplify due to changes at a priming site, or sampling artifacts; the last of these is the most straightforward explanation. 

Paralogy (Table 1, 2–4) can either complicate or clarify phylogenetic relationships. If duplicate copies are retained and are well-sampled (Table 1, 2), paralogy can be detected as intra-genome polymorphism shared among the species that have arisen since the duplication occurred. Well-sampled copies can potentially provide valuable phylogenetic information. Sparse sampling when paralogy is unsuspected (Table 1, 3), or random loss of paralogs after duplication (Table 1, 4), will yield similar patterns that are difficult to interpret [136,137], such as widespread taxon polyphyly, sporadic intra-individual polymorphism, and discordance among gene trees. Paralogy does not appear to have major effects in the present analyses, although it is difficult to entirely rule it out [137], especially for the GBSSI data set. Initial paralogy assessments for pepC, β-amylase, and GBSSI were based on information about copy number from crop grasses, combined with preliminary phylogenetic analyses of available sequences in Genbank. The primers for the pepC gene [138] were designed to amplify a single copy from the small pepC gene family [139-141], and similarly, the primers for the β-amylase gene [142] target one copy from a small gene family [143]. Neither tree shows evidence of multiple paralogs in the form of shared intra-individual polymorphism or widespread polyphyly. The GBSSI tree is more complicated in terms of taxon polyphyly, unanticipated gene copies (especially in *E. repens*), unexpected relationships among diploid taxa, and occasional intra-individual polymorphism (e.g., *P. spicata*). The GBSSI gene was presumed to be a single copy in the wheat tribe [144] based on earlier studies in crop grasses [145-149]. Recent phylogenetic studies in grasses are generally consistent with a single GBSSI copy [150-154], although duplication was inferred in *Spartina* [155]. Based on the general consensus among existing studies, and the lack of the characteristic pattern of shared intra-individual polymorphisms on the Triticeae GBSSI tree that would indicate paralogy, the sequences used to generate the present GBSSI tree are assumed to be orthologs, with the complicated patterns resulting from other phenomena. 

Allopolyploid sequences that lack clear affinities to extant diploids could reflect incomplete sampling among diploids, or that the progenitor is extinct or undiscovered (Table 1, 5). The **Y** genome from the Asian tetraploids is phylogenetically distinct on all three nuclear gene trees (Figures 3, 4, 5a), and represents a case where the diploid progenitor’s lineage is probably either extinct or so rare as to have remained undiscovered. The long-standing interest in the wheat tribe has led to extensive collection efforts of wild representatives throughout the world over many decades. Throughout numerous cytogenetic studies [66,68,71-74,76-78,98-101] and subsequent phylogenetic analyses [40,102,133], no diploid **Y**-genome species has ever been confirmed; the **Y** genome is a distinct evolutionary lineage known only as a component of certain allopolyploid genomes. 

A second case of a “missing diploid” involves the phylogenetically distinct unknown (“UK”) sequence clade from the hexaploid *E. repens*. It is present on all three nuclear gene trees, suggesting that it represents an entire genome of unknown origin, rather than introgression of a limited number of loci. It is widespread within *E. repens*, appearing in individuals from its native Eurasian range [52] and in introduced plants from widely-separated sites in the United States [113]. In contrast to the well-studied **Y** genome from Asian *Elymus*, the “UK” genome is known only from recent phylogenetic analyses of *E. repens*, so it is more difficult to determine whether the missing diploid is best explained as a sampling artifact vs. an extinct or undiscovered lineage.

If similar polyploid combinations arise recurrently (Table 1, 6), polyploid alleles can be polyphyletic within diploid donor clades. Given several major assumptions – adequate phylogenetic resolution, no prior introgression among donor diploids, no subsequent introgression among polyploids, and no homoeolog loss – each event will potentially result in a separate clade of polyploid sequences arising from within each diploid donor clade. Because entire genomes are involved, recurrent origins should simultaneously affect multiple nuclear gene trees in similar ways. While the design the present study precludes detection of multiple origins from within the same species, recurrent origins of the broader **StStHH** or **StStYY** combinations *are* potentially detectable, because there is enough variation within *Pseudoroegneria* (**St**) and *Hordeum* (**H**) to provide phylogenetic signal within each clade (Figures 2–5). There are several cases where polyploid homolog groups are in fact polyphyletic, including the **St** sequences of the **StStYY** species (Figures 3, 4, 5a) and the North American **StStHH** species (Figures 4, 5a); and the **H** sequences of the North American and Eurasian **StStHH** species (Figure 5b) and of *E. repens* (Figures 3, 4, 5b). However, although polyphyly is seen across multiple trees in some of these cases, the specific polyphyletic patterns are not shared among the trees; thus, the patterns look more consistent with multiple independent introgression events than with recurrent **StStHH** or **StStYY** origins (although subsequent gene exchange among the lineages could obscure shared patterns that would indicate multiple origins).

 Introgression among species (Table 1, 7–11) can lead to tree-specific effects and among-tree incongruence. Few of the phylogenetic patterns observed here are clearly attributable to introgression within the provisional species groups (Table 1, 7, 8). For example, in the **Y**-genome clade of the **StStYY** group, within-species samples (*E. abolinii*, *E. antiquus*, *E. caucasicus*, *E. ciliaris*, and *E. semicostatus*) are either monophyletic or consistent with monophyly; gene exchange among **StStYY** species would disrupt this. The **St** sequences from the **StStYY** group are more complicated in that they are broadly polyphyletic on all three trees, but this broad pattern suggests introgression from outside of the **StStYY** group, rather than among species within it. Similarly, placement of the **StStHH** species reflects among-group introgression involving β-amylase (Figure 4) and GBSSI (Figure 5), but there is no clear evidence of within-group gene exchange. While there is too little resolution to address gene exchange among the **StStHH** species in detail, the consistency of certain relationships across trees (*E. canadensis*, *E. hystrix*, *E. riparius*, and *E. virginicus* **H** sequences; *E. brachyaristatus* and *E. sibiricus* **St** sequences) speaks against extensive interspecific gene exchange among the **StStHH** species. *Elymus mutabilis* represents a possible exception; its placement relative to *E. caninus*, *E. dentatus*, and *E. sibiricus* hints at a history of either within-group gene exchange or incomplete lineage sorting.

 Introgression from taxa outside of a polyploid group (Table 1, 9–11) will result in polyphyly of a polyploid’s homoeologs coupled with incongruence among the gene trees, which is a widespread combination of phenomena here. For example, the **St** sequences of the Asian **StStYY** species are polyphyletic on all three nuclear trees (Figure 3, 4, 5a), most dramatically so on the GBSSI tree (Figure 5a), and the details of the patterns differ among all three trees. In addition, while the pepC tree (Figure 3) points to the Eurasian *P. strigosa* as their potential progenitor, the β-amylase tree (Figure 4) instead places the North American species *P. spicata* as their closest diploid relative (the GBSSI tree is equivocal). Furthermore, the donor genus *Pseudoroegneria* is itself polyphyletic on the GBSSI tree (Figure 5a), and *E. antiquus* shares an anomalous allele with *P. libanotica* and *P. tauri*, whether through a separate polyploidization event or through introgression from *Pseudoroegneria* into *E. antiquus*. The broad polyphyly of these sequences within trees, along with their conflicting placement among trees, highlight the acquisition of alleles from outside the **StStYY** group through introgression. It appears to be a one-way process, in that no **Y**-genome sequences appear in any other species. 

 Within the **H**-genome clade, the Eurasian **StStHH** species show little differentiation on the pepC and β-amylase trees (Figures 3, 4), but are widely polyphyletic on the GBSSI tree (Figure 5b). The *E. repens* **H** homoeologs are polyphyletic on all three nuclear trees, even though its chloroplast sequences (Figure 2) and most of its other nuclear homoeologs are either monophyletic or consistent with monophyly. Both of these cases can be explained by multiple instances of introgression from *Hordeum* into *Elymus*. Exchange between *Hordeum* and *Elymus* might, but need not, involve gene exchange across ploidy levels, as there are numerous tetraploid and hexaploid *Hordeum* species [58]. 

*Elymus repens* not only exhibits introgression from *Hordeum*, but from several other sources. A *Taeniatherum*-like clade of *E. repens* sequences appears only on the GBSSI tree (Figure 5a), and is thus probably a result of introgression. It is found in native accessions [52] as well as the introduced U. S. accessions analyzed here. Furthermore, the placement of *Taeniatherum* is itself anomalous on the GBSSI tree, away from its usual position in or near the *Triticum/Aegilops* group (Figures 2–4), so *Taeniatherum*’s GBSSI copy was itself probably gained through introgression. Whether these two events were independent or sequential is not clear. The more distant Poeae-like GBSSI copy (Figure 6), as well as apparent *Bromus*- and *Panicum*-like ITS sequences [52] reveal this species to be a highly complex entity, with a history of acquiring genetic material from distant sources.

 Finally, introgression among diploid species (Table 1, 11) can lead to gene-tree incongruence involving both diploids and their derived polyploids. One possible case was mentioned above: *P. tauri* and *P. libanotica* are outside the main **St** sequence group on the GBSSI gene (Figure 5a), along with the **StStYY** species *E. antiquus*. A possible scenario is that *P. tauri* and *P. libanotica* are misplaced due to the acquisition a foreign GBSSI allele, which was then passed to *E. antiquus* through hybridization, or during an independent polyploidization event. Another possible case involves the diphyletic *P. spicata* on the GBSSI tree: one *P. spicata* allele (6c) is found in a largely Eurasian clade with *P. strigosa* and a mix of Eurasian and North American **StStHH** species. Thus, *P. spicata* might have acquired a Eurasian **St** allele through introgression, and passed it into the North American **StStHH** group through hybridization or during polyploidization.

 Different phenomena have different effects – on polyploids vs. diploids, and on single gene trees vs. multiple trees – so it is reasonable to favor some processes over others based on gene tree topologies. However, different processes can produce very similar effects, and when they act simultaneously, they can alter or mask one another’s effects. Thus, as the discussion above illustrates, the interpretation of trees in terms of individual evolutionary processes is speculative in all but the most straightforward situations. 

### Polyploid Classification

Classification of stable reticulate taxa creates a logical conundrum in a hierarchical system [156-159], even for the most straightforward allopolyploid taxa arising from a single origin, and with no subsequent introgression. The recognition of an allotetraploid lineage as a separate named genus makes sense in some ways – from a population standpoint, species that arise through diversification from a single polyploid ancestor can be defined as a monophyletic group (e.g., as applied by [6,22]). On the other hand, recognition of an allopolyploid lineage as a taxon renders both of the originating groups paraphyletic [160,161]. For example, even if the **StStHH** tetraploids arose very simply via a single origin, their recognition as *Elymus* leaves *Pseudoroegneria* and *Hordeum* paraphyletic. As more polyploids are considered (e.g., the **StStYY** group and *E. repens*), the problems with circumscribing *Pseudoroegneria* and *Hordeum* increase. Mindell [162], using holobiont phylogeny as an example of reticulate evolution, recommended expanding the definition of groups from which a composite taxon is derived. Thus, in his example, green plant chloroplasts would be properly classified within the *Cyanobacteria*. Applying this approach to an intergeneric polyploid lineage – separately classifying the **St** and **H** genomes of *Elymus* within their ancestral genera *Pseudoroegneria* and *Hordeum* – has some logical appeal, especially insofar as the **St** and **H** homoeologs do not pair at meiosis, and each genome’s integrity is maintained within the *Elymus* polyploid nucleus. This would also open the door to naming the **Y**-genome clade, even though it is only known to exist as a component within larger allopolyploid genomes. Overall, however, this approach to classification is unintuitive and impractical when applied to individual plants and taxa. A simpler approach would be to classify the polyploids and all of their progenitors as a single genus, thus avoiding the entire problem of paraphyletic genera. Ultimately, this would result in defining most or all of the tribe as a single genus, which is not without precedent [163], although this would do little more than push the same classification issues to the subgeneric level. Furthermore, while a single-genus solution might reasonably be proposed for a more obscure group, it would be unlikely to gain acceptance for this tribe which, due to its economic importance, has undergone a long history of close scrutiny [164] and intense taxonomic focus [165]. 

## Conclusion

**Figure 8:**
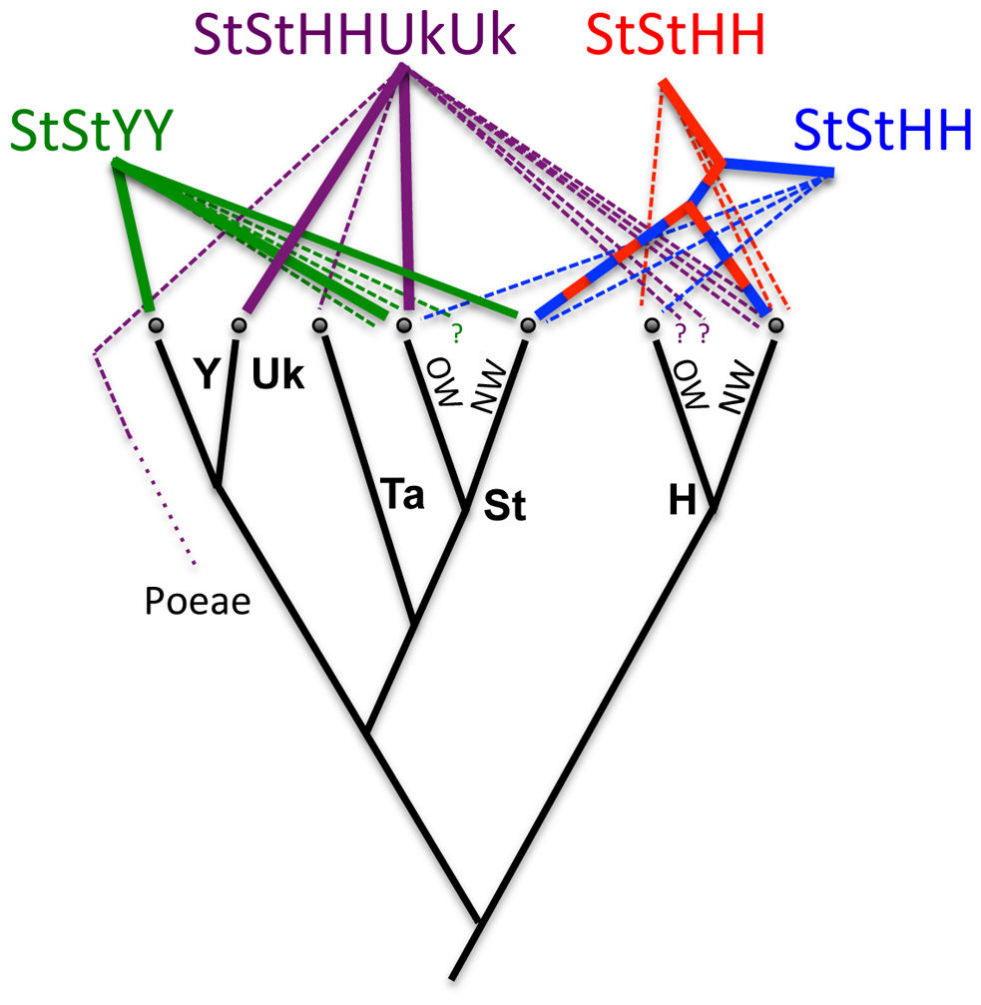
Summary diagram of *Elymus* origins. Unbroken lines represent major contributions, and dotted lines show minor contributions. Colors follow Figures 1–7. The simple bifurcating division of *Pseudoroegneria* (St) and *Hordeum* (**H**) progenitor species into Old World (OW) and New World (NW) lineages is used for illustrative purposes and does not reflect their true phylogenetic complexity, particularly within *Hordeum*.

Ultimately, the results and discussion presented here might have done less to clarify the history of *Elymus* than to highlight the difficulty of developing a step-by-step, gene-by-gene reconstruction of the evolutionary events leading to the present situation (Figure 8). This is appropriate to the final impression I hope to leave – that of a genetically tangled set of lineages that are evolutionarily distinct, but are neither entirely separable from one another, nor fully cohesive individually.

## Supporting Information

Dataset S1 **Chloroplast trnT/L/F genes and spacers, and rpoA gene sequences (see Figure 2).**
(TXT)

Dataset S2 **Phosphoenolpyruvate carboxylase partial gene sequences (see Figure 3).**
(TXT)

Dataset S3 **Beta-amylase partial gene sequences (see Figure 4).**
(TXT)

Dataset S4 **Granule-bound starch synthase partial gene sequences (see Figure 5).**
(TXT)

Dataset S5 **Granule-bound starch synthase partial gene sequences with introns excluded (see Figure 6).**
(TXT)

Table S1 **List of taxa, collection information, and Genbank accession numbers for sequences analyzed for Figures 2–5.**
(PDF)

Table S2 **Sequences used in GBSSI exon analysis (Figure 6).**
(PDF)

Table S3 **Datasets, partitions where applicable, models selected under AIC criterion, and parameters estimated on the best ML tree.**
(PDF)
