# Supplementary material for: Phylogeny of a Genomically Diverse Group of Elymus (Poaceae) Allopolyploids Reveals Multiple Levels of Reticulation
Source: PLoS One. 2013 Nov 1;8(11):e78449. doi: 10.1371/journal.pone.0078449 (PMC3840256; doi:10.1371/journal.pone.0078449)
Supplement: Table S1 — List of taxa, collection information, and Genbank accession numbers for sequences analyzed for Figures 2–5. (PDF) [file pone.0078449.s006.pdf]

**Table S1. List of taxa, collection information, and Genbank accession numbers for sequences analyzed for Figures 2–5.**

| Eurasian *Elymus*–StStHH | Desig[1] | Voucher | Accession | Origin | Genbank pepC[2] | Genbank β-amy | Genbank GBSSI | Genbank rpoA | Genbank trnT-F |
|---|---|---|---|---|---|---|---|---|---|
| *Elymus breviaristatus* Keng ex Keng f. | 1 | ID | PI 499411 | China | b-HM035290-St<br>e-HM035291-H | b-HM035223-St<br>a-HM035224-H | a-HM035268-St<br>b-HM035269-H | KF600660 | KF600684 |
| *Elymus caninus* (L.) L. | 1 | ID | PI 314205 | Uzbekistan | h-HM035292-St<br>b-HM035293-H | a-HM035225-St<br>d-HM035226-H | n-DQ159325-St<br>a-DQ159324-H | KF600661 | KF600685 |
| *Elymus caninus* | 2 | ID | PI 314612 | Kazakhstan | a-HM035294-St<br>d-HM035295-H<br>i-HM035296-H | d-HM035227-St<br>c-HM035228-H | b-HM035270-St<br>a-HM035271-H | KF600662 | KF600686 |
| *Elymus caninus* | 4 | ID | PI 499413 | China | d-HM035297-St<br>f-HM035298-H | d-HM035229-St<br>b-HM035230-H | a-HM035272-St<br>b-HM035273-H | KF600663 | KF600687 |
| *Elymus caninus* | 5 | ID | PI 531571 | Poland | e-HM035299-St<br>g-HM035300-H | d-HM035231-St<br>e-HM035232-H | a-HM035274-St<br>b-HM035275-H | KF600664 | KF600688 |
| *Elymus dentatus* (Hook.f.) Tzvelev | 1 | ID | PI 628702 | Russia | d-HM035301-St<br>c-HM035302-H<br>e-HM035303-H | a-HM035233-St<br>d-HM035234-H | a-DQ159328-St<br>b-DQ159329-H | DQ159334 | DQ159290 |
| *Elymus dentatus* | 2 | ID | PI 531599 | Pakistan | a-HM035304-St<br>b-HM035305-H | i-HM035235-St<br>a-HM035236-H | a-HM035276-St<br>b-HM035277-H | KF600667 | KF600691 |
| *Elymus mutabilis* (Drobow) Tzvelev | 1 | ID | PI 628704 | Russia | NR-St[3]<br>a-HM035306-H<br>i-HM035307-H | a-HM035237-St<br>c-HM035238-H | c-DQ159331-St<br>a-DQ159330-H | DQ159335 | DQ159291 |
| *Elymus mutabilis* | 2 | ID | PI 499449 | China | h-HM035308-St<br>a-HM035309-H<br>f-HM035310-H | h-HM035239-St<br>c-HM035240-H | a-HM035278-St<br>b-HM035279-H | KF600670 | KF600694 |
| *Elymus sibiricus* L. | 1 | ID | PI 628699 | Russia | b-HM035311-St<br>a-HM035312-H | NR-St<br>b-HM035241-H | b-HM035280-St<br>a-HM035281-H | KF600673 | KF600697 |
| *Elymus sibiricus* | 3 | ID | PI 499461 | China | d-HM035313-St<br>a-HM035314-H | g-HM035242-St<br>b-HM035243-H | a-HM035282-St<br>b-HM035283-H | KF600674 | KF600698 |

| North American *Elymus*–StStHH | Desig[1] | Voucher | Accession | Origin | Genbank pepC | Genbank β-amy | Genbank GBSSI | Genbank rpoA | Genbank trnT-F |
|---|---|---|---|---|---|---|---|---|---|
| *Elymus canadensis* L. | 1 | ID | Barkworth 97-86 | USA | NS[4] | NS | NS | AY115928 | AF519131 |
| *Elymus canadensis* | 2 | ID | PI 578675 | USA | b-HM035315-St<br>a-AY553243-H | NS | NS | AY115929 | AF519132 |
| *Elymus canadensis* | 4 | ID | PI 531568 | USA | b-AY553248-St<br>a-AY553242-H | a-HM035244-St<br>d-HM035245-H | c-HM035284-St<br>a-HM035285-H | AY115930 | AF519133 |
| *Elymus elymoides* (Raf.) Swezey | 1 | ID | PI 531606 | USA | b-AY553249-St<br>a-AY553244-H | a-HM035246-St<br>c-HM035247-H | a2-AY010992-St<br>d2-AY010965-H | AY115932 | AF519135 |
| *Elymus glaucus* Buckley | 4 | ID | RJMG 130 | USA | NS | NS | a-AY01097-St<br>b-AY010966-H | AY115933 | AF519136 |
| *Elymus glaucus* | 6 | ID | W6 10215 | USA | b-AY553250-St<br>e-HM035316-H | NR-St<br>a-HM035248-H | b-AY010980-St<br>a-AY010967-H | AY115934 | AF519137 |
| *Elymus glaucus* | 7 | ID | PI 593652 | USA | NS | NS | NS | AY115935 | AF519138 |
| *Elymus hystrix* L. | 1 | ID | Barkworth 97-87 | USA | b-AY553251-St<br>a-AY553245-H | NR-St<br>a-HM035249-H | a-AY010982-St<br>d-HM035286-H | AY115936 | AF519139 |
| *Elymus lanceolatus* (Scribn. & J.G.Sm.) D.R.Dewey | 1 | ID | PI 632507 | USA | NS | c-HM035250-St<br><br>a-HM035251-H | a-AY010993-St<br>d-AY010984-St<br>aa-AY010969-H | AY115937 | NS |
| *Elymus lanceolatus* | 2 | ID | PI 632506 | USA | NS | a-HM035252-St<br><br>h-HM035253-H | c-AY010985-St<br>d-AY010994-St<br>a-AY010970-H | NS | NS |
| *Elymus lanceolatus* | 3 | ID | PI 531623 | USA | b-AY553252-St<br>a-AY553246-H | NS | NS | AY115938 | AF519140 |
| *Elymus riparius* Wiegand | 1 | ID | RJMG 160 | USA | NS | a-HM035254-St<br>b-HM035255-H | NR-St<br>a-AY010971-H | NS | NS |
| *Elymus trachycaulus* (Link) Gould ex Shinners | 1 | ID | PI 372500 | Canada | e-HM035317-St<br>a-HM035318-H | a-HM035256-St<br>b-HM035257-H | b1-AY010986-St<br>a1-AY010972-H | AY115939 | AF519141 |
| *Elymus trachycaulus* | 3 | ID | PI 452446 | Canada | NS | NS | d-AY010987-St<br>a-AY010973-H<br>b-AY010974-H | AY115940 | AF519142 |

| | | | | | | | | | |
|---|---|---|---|---|---|---|---|---|---|
| *Elymus virginicus* L. | 1 | ID | PI436945 | USA | NS | NS | NS | AY115941 | AF519143 |
| *Elymus virginicus* | 2 | ID | PI490361 | USA | NS | NS | NS | AY115942 | AF519144 |
| *Elymus virginicus* | 4 | ID | RJMG 161 | USA | NR-St<br>a-HM035319-H | NR-St<br>a-HM035258-H | d-AY010995-St<br>a-AY010975-H | NS | NS |
| *Elymus virginicus* | 6 | ID | RJMG163 | USA | NS | NS | NS | AY115943 | AF519145 |
| *Elymus virginicus* | 9 | ID | RJMG 168 | USA | d-HM035320-St<br>a-HM035321-H | a-HM035259-St<br>e-HM035260-H | a-AY010989-St<br>b-AY010976-H | NS | NS |
| *Elymus wawawaiensis* J.R.Carlson & Barkworth | 1 | ID | PI 285272 | USA | NS | a-HM035261-St<br>d-HM035262-H | c-AY010996-St<br>a-AY010977-H | AY115944 | AF519146 |
| *Elymus wawawaiensis* | 3 | ID | PI 598812 | USA | b-AY553253-St<br>a-AY553247-H | c-HM035263-St<br>b-HM035264-H | a-AY010990-St<br>b-AY010997-St<br>d-AY010978-H | AY115945 | AF519147 |

| **Asian *Elymus*–StStYY** | **Desig[1]** | **Voucher** | **Accession** | **Origin** | **Genbank pepC** | **Genbank β-amy** | **Genbank GBSSI** | **Genbank rpoA** | **Genbank trnT-F** |
|---|---|---|---|---|---|---|---|---|---|
| *Elymus abolinii* (Drobow) Tzvelev | 1 | ID | PI 531555 | China | b-GQ844927-St<br>a-GQ844928-Y | b-GQ847678-St<br>a-GQ847679-Y | a-DQ159322-St<br>b-DQ159323-Y | DQ159332 | DQ159288 |
| *Elymus abolinii* | 2 | ID | PI 531557 | Estonia | b-GQ844929-St<br>i-GQ844930-Y | a-GQ847680-St<br>NR-Y | k-GQ847708-St<br>a-GQ847709-Y<br>e-GQ847710-Y | NS | NS |
| *Elymus antiquus* (Nevski) Tzvelev | 1 | ID | PI 632564 | China | g-GQ844931-St<br>a-GQ844932-Y | a-GQ847681-St<br>f-GQ847682-Y | a-GQ847711-St<br>b-GQ847712-Y | KF600659 | KF600683 |
| *Elymus antiquus* | 2 | ID | PI 564958 | China | a-GQ844933-St<br>c-GQ844934-Y | a-GQ847683-St<br>e-GQ847684-Y | g-GQ847713-St<br>a-GQ847714-Y | NS | NS |
| *Elymus antiquus* | 3 | ID | PI 619528 | China | b-GQ844935-St<br>d-GQ844936-St<br>c-GQ844937-Y | a-GQ847685-St<br>e-GQ847686-Y | b-GQ847715-St<br>a-GQ847716-Y | NS | NS |
| *Elymus antiquus* | 4 | ID | PI 564957 | China | c-GQ844938-St<br>h-GQ844939-Y | d-GQ847687-St<br>a-GQ847688-Y | b-GQ847717-St<br>a-GQ847718-Y | NS | NS |
| *Elymus caucasicus* (K.Koch) Tzvelev | 1 | ID | PI 531573 | Estonia | a-GQ844940-St<br>d-GQ844941-Y | b-GQ847689-St<br>a-GQ847690-Y | NR-St<br>a-GQ847719-Y<br>b-GQ847720-Y | DQ159333 | DQ159289 |

| | | | | | | | | | |
|---|---|---|---|---|---|---|---|---|---|
| *Elymus ciliaris* (Trin.) Tzvelev | 1 | GH | PI 531575 | China | b-GQ844942-St<br>c-GQ844943-Y<br>h-GQ844944-Y | g-GQ847691-St<br>h-GQ847692-Y | g-DQ159327-St<br>b-DQ159326-Y | AY115931 | AF519134 |
| *Elymus ciliaris* | 2 | ID | PI 531577 | Japan | d-GQ844945-St<br>a-GQ844946-Y | f-GQ847693-St<br>i-GQ847694-Y | a-GQ847721-St<br>b-GQ847722-Y | KF600665 | KF600689 |
| *Elymus ciliaris* | 5 | ID | PI 531576 | Estonia | a-GQ844947-St<br>c-GQ844948-Y | e-GQ847695-St<br>a-GQ847696-Y | a-GQ847723-St<br>b-GQ847724-St<br>c-GQ847725-Y | KF600666 | KF600690 |
| *Elymus gmelinii* (Ledeb.) Tzvelev | 1 | ID | PI 499447 | China | NR-St<br>a-GQ844949-Y | e-GQ847697-St<br>f-GQ847698-Y | a-GQ847726-St<br>b-GQ847727-Y | KF600668 | KF600692 |
| *Elymus longearistatus* (Boiss.) Tzvelev | 1 | ID | PI 401277 | Iran | c-GQ844950-St<br>aa-GQ84451-Y | NR-St<br>e-GQ847699-Y | NR-St<br>d-GQ847728-Y | KF600669 | KF600693 |
| *Elymus nevskii* Tzvelev | 1 | ID | PI 314620 | Kazakhstan | a-GQ844952-St<br>b-GQ844953-Y | k-GQ847700-St<br>b-GQ847701-Y | a-GQ847729-St<br>b-GQ847730-Y | KF600671 | KF600695 |
| *Elymus pendulinus* (Nevski) Tzvelev | 1 | ID | PI 499452 | China | a-GQ844954-St<br>b-GQ844955-Y | i-GQ847702-St<br>b-GQ847703-Y | a-GQ847731-St<br>b-GQ847732-Y | KF600672 | KF600696 |
| *Elymus semicostatus* (Nees ex Steud.) Melderis | 1 | ID | PI 271522 | India | d-GQ844956-St<br>a-GQ844957-Y | c-GQ847704-St<br>a-GQ847705-Y | f-GQ847733-St<br>d-GQ847734-Y | NS | NS |
| *Elymus semicostatus* | 2 | ID | PI 207453 | Afghanistan | a-GQ844958-St<br>NR-Y | NR-St<br>a-GQ847706-Y | a-GQ847735-St<br>e-GQ847736-Y | NS | NS |

| *Elymus repens*–StStHHUkUk | Desig[1] | Voucher | Accession | Origin | Genbank pepC | Genbank β-amy | Genbank GBSSI | Genbank rpoA | Genbank trnT-F |
|---|---|---|---|---|---|---|---|---|---|
| *Elymus repens* (L.) Gould | 1 | ID | RJMG119 | USA | g-EU282271-St | d-EU282239-St | f[6]-AY360849-St | AY362780 | AY362786 |
| | | | | | | | c-AY360826-St | | |
| | | | | | a-EU282269-H | q-EU282240-H | e-AY360827-H | | |
| | | | | | q-EU282272-H | r-EU282241-H | | | |
| | | | | | b-EU282270-Uk | c-EU282238-Uk | q-AY360828-Uk1 | | |
| | | | | | NH[5] | NH | a1-AY360825-T | | |
| | | | | | NH | NH | g[6]-AY360858-Uk2 | | |
| *Elymus repens* | 2 | ID | RJMG122 | USA | NR-St | i-EU282243-St | g[6]-AY360850-St | AY362781 | AY362787 |
| | | | | | b-EU282274-H | k-EU282244-H | NR-H | | |
| | | | | | h-EU282275-H | | | | |
| | | | | | a-EU282273-Uk | a-EU282242-Uk | aa-AY360830-Uk1 | | |
| | | | | | | | cc-AY360832-Uk1 | | |
| | | | | | NH | NH | bb-AY360831-T | | |
| | | | | | NH | NH | hh-AY360829-T | | |
| | | | | | NH | NH | h[6]-AY360859-Uk2 | | |
| *Elymus repens* | 3 | ID | RJMG131 | USA | d-EU282276-St | a-EU282245-St | f[6]-AY360851-St | AY362782 | AY362788 |
| | | | | | f-EU282277-St | | | | |
| | | | | | h-EU282278-H | d-EU282247-H | NR-H | | |
| | | | | | | l-EU282248-H | | | |
| | | | | | i-EU282279-Uk | c-EU282246-Uk | h[6]-AY360852-Uk1 | | |
| | | | | | NH | NH | a-AY360833-T | | |
| | | | | | NH | NH | aa-AY360834-T | | |
| | | | | | NH | NH | dd-AY360835-T | | |
| | | | | | NH | NH | g[6]-AY360860-Uk2 | | |
| *Elymus repens* | 4 | ID | RJMG159 | USA | e-EU282281-St | b-EU282250-St | NR-St | AY362783 | AY362789 |
| | | | | | q-EU282283-St | | | | |
| | | | | | m-EU282282-H | x-EU282251-H | dd-AY360839-H | | |
| | | | | | c-EU282280-Uk | a-EU282249-Uk | a-AY360836-Uk1 | | |
| | | | | | | | b[6]-AY360853-Uk1 | | |
| | | | | | | | i[6]-AY360854-Uk1 | | |
| | | | | | NH | NH | aa-AY360838-T | | |
| | | | | | NH | NH | t[6]-AY360837-T | | |

| | | | | | | | | | |
|---|---|---|---|---|---|---|---|---|---|
| *Elymus repens* | 5 | ID | RJMG166 | USA | i-EU282288-St | b-EU282253-St | NR-St | AY362784 | AY362790 |
| | | | | | l-EU282289-St | | | | |
| | | | | | a-EU282284-H | a-EU282252-H | j[6]-AY360855-H | | |
| | | | | | b-EU282285-H | | | | |
| | | | | | c-EU282286-H | | | | |
| | | | | | e-EU282287-Uk | c-EU282254-Uk | k-AY360840-Uk1 | | |
| | | | | | NH | NH | cc-AY360841-T | | |
| | | | | | NH | NH | dd-AY360842-T | | |
| | | | | | NH | NH | b[6]-AY360861-Uk2 | | |
| | | | | | NH | NH | hh-AY360843-Uk2 | | |
| *Elymus repens* | 6 | ID | RJMG167 | USA | d-EU282291-St | NS | a[6]-AY360856-St | AY362785 | AY362791 |
| | | | | | | | g[6]-AY360857-St | | |
| | | | | | c-EU282290-H | | dd-AY360845-H | | |
| | | | | | e-EU282292-Uk | | hh-AY360846-Uk1 | | |
| | | | | | NH | NH | a2-AY360844-T | | |
| **Monogenomic Triticeae** | **Desig[1]** | **Voucher** | **Accession** | **Origin** | **Genbank pepC** | **Genbank β-amy** | **Genbank GBSSI** | **Genbank rpoA** | **Genbank trnT-F** |
| *Aegilops bicornis* (Forsskål) Jaub. & Spach | 1 | GH | L. Morrison s.n. | | NS | AY821686 | NS | NS | NS |
| *Aegilops caudata* L. | 1 | GH | G 758 | | NS | a-AY821687 | AF079262 | AY115908 | AF519111 |
| | | | | | | b-AY821688 | | | |
| | | | | | | c-AY821689 | | | |
| *Aegilops comosa* Sibth. & Sm. | 1 | GH | G 602 | | AY553236 | a-AY821690 | NS | NS | NS |
| | | | | | | g-AY821696 | | | |
| *Aegilops speltoides* Tausch | 1 | GH | L. Morrison s.n. | | NS | KF600658 | AF079267 | AY115909 | AF519112 |
| *Aegilops tauschii* Coss. | 1 | GH | L. Morrison s.n. | | NS | AY821695 | AF079268 | AY115910 | AF519113 |
| *Aegilops uniaristata* Vis. | 1 | GH | G 1297 | | NS | AY821691 | AF079270 | AY115911 | AF519114 |
| *Agropyron cristatum* (L.) Gaertn. | 1 | GH | PI 279802 | Canada | AY553237 | AY821697 | AF079271 | AY115912 | AF519115 |
| *Agropyron cristatum* | 2 | GH | PI 281862 | Germany | NS | NS | AY011002 | AY115913 | AF519116 |
| *Agropyron mongolicum* Keng | 1 | GH | D 2774 | China | NS | NS | AY011003 | AY115914 | AF519117 |
| *Australopyrum retrofractum* (Vickery) Á.Löve | 1 | GH | PI 533013 | Australia | NS | AY821692 | AF079272 | AY115915 | AF519118 |
| *Australopyrum velutinum* (Nees) B.K.Simon | 1 | GH | D 2873-2878 | Australia | AY553238 | AY821693 | AY011004 | AY115916 | AF519119 |
| *Crithopsis delileana* (Schult.) Roshev. | 1 | GH | H 5562 | Jordan | NS | AY821694 | GQ847707 | NS | NS |

| | | | | | | | | | |
|---|---|---|---|---|---|---|---|---|---|
| *Dasypyrum villosum* (L.) Candargy | 1 | GH | PI 251478 | Turkey | NS | AY821698 | AF079274 | AY115925 | AF519128 |
| *Dasypyrum villosum* | 2 | GH | PI 470279 | Turkey | NS | AY821699 | NS | AY115926 | AF519129 |
| *Dasypyrum villosum* | 3 | GH | D 2990 | Australia | AY553240 | NS | NS | NS | NS |
| *Eremopyrum bonaepartis* (Spreng.) Nevski | 1 | GH | H 5554 | Iran | NS | AY821700 | AY011005 | AY115946 | AF519148 |
| *Eremopyrum bonaepartis* | 2 | GH | H 5569 | | NS | NS | NS | AY115947 | AF519149 |
| *Eremopyrum distans* (K.Koch) Nevski | 1 | GH | H 5552 | Iran | NS | AY821701 | AY011006 | AY115948 | AF519150 |
| *Eremopyrum orientale* (L.) Jaub. & Spach | 1 | GH | H 5555 | Iran | AY553254 | AY821702 | AY011007 | AY115949 | AF519151 |
| *Henrardia persica* (Boiss.) C.E.Hubb. | 1 | GH | H 5556 | Iran | NS | AY821703 | AF079276 | AY115950 | AF519152 |
| *Heteranthelium piliferum* (Banks & Sol.) Hochst. | 1 | GH | PI 402352 | Iran | AY553255 | AY821704 | AF079277 | AY115951 | AF519153 |
| *Hordeum bogdanii* Wilensky | 1 | ID | PI 531762 | Tajikistan | NS | GQ847675 | EU282316 | NS | NS |
| *Hordeum bogdanii* | 2 | ID | PI 531760 | China | EU282293 | EU282255 | EU282317 | NS | NS |
| *Hordeum brachyantherum* Nevski | 1 | GH | PI 531764 | USA | NS | NS | NS | AY115917 | AF519120 |
| *Hordeum brevisubulatum* (Trin.) Link | 1 | GH | PI 401387 | Iran | NS | a-AY821705<br>d-AY821712 | AY010961 | AY115918 | AF519121 |
| *Hordeum brevisubulatum* | 2 | GH | PI 401390 | Iran | NS | AY821713 | AY010964 | NS | NS |
| *Hordeum bulbosum* L. | 1 | GH | PI 440417 | Kazakhstan | a-EU282294<br>b-EU282295<br>c-EU282296 | AY821706 | AY010962 | AY115919 | AF519122 |
| *Hordeum californicum* Covas & Stebbins | 1 | GH | MA-138-1-40 | USA | AY553256 | AY821707 | AF079273 | NS | KF600706 |
| *Hordeum chilense* Roem. & Schult. | 1 | ID | PI 531781 | Argentina | EU282297 | - | EU282318 | NS | NS |
| *Hordeum jubatum* L. | 1 | ID | RJMG 106 | USA | a-AY553257<br>f-HM035287 | a-AY821708<br>b-AY821709 | a-AY010963<br>d-HM035266 | AY115920 | AF519123 |
| *Hordeum jubatum* | 2 | ID | RJMG 134 | USA | f-HM035288<br>i-HM035289 | c-AY821710<br>a-AY821711 | f-HM035265<br>m-HM035267 | NS | NS |
| *Hordeum marinum* Huds. | 1 | ID | PI 304346 | USA | AY553258 | a-EU282256<br>b-EU282257 | AY010959 | AY115921 | AF519124 |
| *Hordeum marinum* | 2 | ID | PI 401364 | Iran | NS | NS | NS | NS | KF600707 |
| *Hordeum marinum* | 3 | ID | PI 304347 | England | EU282298 | EU282258 | EU282319 | NS | NS |
| *Hordeum murinum* L. | 1 | ID | PI 247054 | USA | a-EU282299<br>b-EU282300 | EU282259 | EU282320 | AY115922 | AF519125 |
| *Hordeum murinum* | 2 | ID | CIho 15683 | USA | AY553259 | EU282260 | AY010960 | AY115923 | AF519126 |

| | | | | | | | | | |
|---|---|---|---|---|---|---|---|---|---|
| *Hordeum pusillum* Nutt. | 2 | ID | CIho 15654 | USA | EU282301 | EU282261 | EU282321 | AY115924 | AF519127 |
| *Hordeum stenostachys* Godr. | 1 | ID | PI 531791 | Argentina | EU282302 | EU282262 | EU282322 | NS | NS |
| *Hordeum vulgare* L. | 1 | na | | | NS | NS | X07931 | NS | NS |
| *Hordeum vulgare* | 2 | ID | RJMG 107 | USA | AY553260 | EU282263 | NS | NS | KF600708 |
| *Leymus racemosus* ssp. *sabulosus* (M.Bieb.) Tzvelev | 1 | GH | PI 531813 | Estonia | AY553261 | NS | NS | NS | NS |
| *Peridictyon sanctum* (Janka) Seberg, Fred., & Baden | 1 | GH | KJ 248 | Greece | AY553262 | AY821714 | AF079278 | AY115952 | AF519154 |
| *Psathyrostachys fragilis* (Boiss.) Nevski | 1 | GH | PI 343192 | Iran | NS | AY821715 | AF079279 | AY115967 | AF519169 |
| *Psathyrostachys juncea* (Fisch.) Nevski | 1 | GH | PI 206684 | Turkey | NS | AY821716 | AF079280 | AY115968 | AF519170 |
| *Pseudoroegneria libanotica* (Hack.) D.R.Dewey | 1 | GH | PI 228391 | Iran | EU282304 | EU282264 | EU282324 | AY115955 | AF519156 |
| *Pseudoroegneria libanotica* | 3 | GH | PI 228392 | Iran | EU282305 | EU282265 | EU282325 | KF600675 | KF600699 |
| *Pseudoroegneria spicata* (Pursh) Á.Löve subsp. *spicata* | 1 | GH | PI 232117 | USA | NS | AY821717 | AF079281 | NS | NS |
| *Pseudoroegneria spicata* subsp. *inermis* (Scribn. & J.G.Smith) Á.Löve | 2 | GH | PI 236681 | Canada | NS | AY821718 | AY010998 | AY115956 | AF519157 |
| *Pseudoroegneria spicata* subsp. *spicata* | 3 | GH | PI 610986 | USA | AY553263 | NS | AY010999 | AY115957 | AF519158 |
| *Pseudoroegneria spicata* subsp. *spicata* | 4 | GH | D 2844 | USA | AY553264 | AY821719 | AY011000 | AY115958 | AF519159 |
| *Pseudoroegneria spicata* subsp. *spicata* | 6 | ID | RJMG 112 | USA | NS | AY821720 | a-AY011001<br>c-AY010991 | KF600676 | KF600700 |
| *Pseudoroegneria spicata* subsp. *inermis* | 8 | GH | D 2839 | USA | NS | NS | NS | AY115959 | AF519160 |
| *Pseudoroegneria stipifolia* (Czern. ex Nevski) Á.Löve | 2 | GH | PI 313960 | Former USSR | EU282306 | EU282266 | NS | KF600677 | KF600701 |
| *Pseudoroegneria stipifolia* | 3 | GH | PI 531751 | Ukraine | a-EU282307<br>d-EU282308 | AY821721 | NS | KF600678 | KF600702 |
| *Pseudoroegneria strigosa* (M.Bieb.) Á.Löve | 1 | GH | PI 499637 | China | b-EU282309<br>d-EU282310 | NS | EU282323 | AY115953 | AF519155 |
| *Pseudoroegneria strigosa* ssp. *aegilopoides* (Drobow) Á.Löve | 2 | GH | PI 531755 | China | EU282311 | EU282267 | AY360823 | AY115954 | KF624612 |
| *Pseudoroegneria tauri* (Boiss. & Balansa) Á.Löve | 1 | GH | PI 380652 | Iran | EU282312 | EU282268 | EU282326 | KF600679 | KF600703 |
| *Pseudoroegneria tauri* | 2 | GH | PI 401319 | Iran | EU282313 | NS | EU282327 | KF600680 | KF600704 |

| | | | | | | | | | |
|---|---|---|---|---|---|---|---|---|---|
| *Pseudoroegneria tauri* | 3 | GH | PI 380644 | Iran | a-EU282314<br>b-EU282315 | NS | - | KF600681 | KF600705 |
| *Secale cereale* L. | 1 | GH | E.Kellogg s.n. | | AY553266 | a-AY821723<br>b-AY821724 | AY011009 | AY115960 | AF519162 |
| *Secale montanum* (C.Presl.) C.Presl. | 1 | GH | PI 440654 | Hungary | NS | NS | AF079282 | AY115961 | AF519161 |
| *Secale montanum* | 2 | GH | T 36554 | | NS | AY821725 | NS | NS | NS |
| *Secale strictum* subsp. *anatolicum* (Boiss.) K.Hammer | 1 | GH | PI 206992 | Turkey | AY553265 | AY821722 | AY011008 | AY115962 | AF519163 |
| *Taeniatherum caput-medusae* (L.) Nevski | 1 | GH | PI 208075 | Turkey | - | AY821726 | AY011010 | AY115963 | AF519164 |
| *Taeniatherum caput-medusae* | 2 | ID | RJMG 189 | USA | AY553268 | AY821727 | b-AY360847<br>e-AY360848 | NS | NS |
| *Taeniatherum caput-medusae* | 3 | ID | PI 314697 | Kazakhstan | NS | AY821728 | NS | NS | NS |
| *Taeniatherum caput-medusae* | 4 | ID | PI 317475 | Afghanistan | NS | AY821729 | NS | NS | NS |
| *Thinopyrum bessarabicum* (Savul. & Rayss) Á.Löve | 1 | GH | PI531711 | Estonia | NS | AY821730 | AF079283 | AY115964 | AF519165 |
| *Thinopyrum elongatum* (Host) D.R.Dewey | 1 | GH | PI 531719 | France | NS | AY821731 | AF079284 | AY115965 | AF519166 |
| *Thinopyrum elongatum* | 2 | ID | RJMG 113 | USA | AY553269 | NS | NS | NS | NS |
| *Thinopyrum scirpeum* (C.Presl.) D.R.Dewey | 1 | GH | PI 531749 | Italy | NS | GQ847676 | AY011011 | AY115966 | AF519167 |
| *Triticum aestivum* L. | 1 | na | | | AJ007705 | NS | NS | NS | NS |
| *Triticum baeoticum* Boiss. | 1 | GH | L.Morrison s.n. | | NS | AY821732 | AF079285 | NS | AF519168 |
| *Triticum monococcum* L. | 1 | GH | PI 221413 | Serbia | NS | AY821733 | NS | NS | NS |
| *Triticum urartu* Tumanian | 1 | GH | L.Morrison s.n. | | NS | GQ847677 | AF079287 | NS | NS |
| *Bromus tectorum* L. | 1 | GH | E.Kellogg s.n. | USA | AY553239 | AY821734 | AY362757 | KF600682 | KF600709 |

[1]Numerical designations distinguish individuals within species, and correspond to the numbers after the taxon names in Figs. 2–6.
[2]Letter designations preceding Genbank accession number distinguish clones from within individuals where applicable, and correspond to the letters following the numerical designations in Figs. 2–6. Genome designations for each sequence following accession numbers were determined based on phylogeny.
[3]NR – sequence expected based on presumed genomic complement but Not Recovered.
[4]NS – gene or region was Not Sequenced for that individual.
[5]NH – there is No Homoeolog of pepC or β-amy corresponding to the T or Uk2 GBSSI homoeologues of *E. repens.*
[6]Shorter GBSSI fragments generated using the waxy K and F primers [6]; indicated by “FK” on Figures 5 and 6.

Sequence sources: KF600659–KF600709, KF624612 [this publication]; X07931 [1]; AF079235–AF079298 [2]; AY010959–AY011011 [3]; AJ007705 [4]; AY115908–AY115966 and AF519111–AF519168 [5]; AY360823–AY360848 and AY362780–AY362791 [6]; AY553236–AY553269 [7]; AY821686–AY821734 [8]; DQ159288–DQ159335 [9]; EU282269–EU282327 [10]; GQ844970–GQ844958 and GQ847675–GQ847736 [11]; HM035223–HM035321 [12].


1. Rohde W, Becker D, Salamini F (1988) Structural analysis of the waxy locus from *Hordeum vulgare*. Nucleic Acids Research 16: 7185–7186.
2. Mason-Gamer RJ, Weil CF, Kellogg EA (1998) Granule-bound starch synthase: structure, function, and phylogenetic utility. Molecular Biology and Evolution 15: 1658–1673.
3. Mason-Gamer RJ (2001) Origin of North American species of *Elymus* (Poaceae: Triticeae) allotetraploids based on granule-bound starch synthase gene sequences. Systematic Botany 26: 757–768.
4. González MC, Echevarría C, Vidal J, Cejudo FJ (2002) Isolation and characterization of a wheat phosphoenolpyruvate carboxylase gene. Modelling of the encoded protein. Plant Science 162: 233–238.
5. Mason-Gamer RJ, Orme NL, Anderson CM (2002) Phylogenetic analysis of North American *Elymus* and the monogenomic Triticeae (Poaceae) using three chloroplast DNA data sets. Genome 45: 991–1002.
6. Mason-Gamer RJ (2004) Reticulate evolution, introgression, and intertribal gene capture in an allohexaploid grass. Systematic Biology 53: 25–37.
7. Helfgott DM, Mason-Gamer RJ (2004) The evolution of North American *Elymus* (Triticeae, Poaceae) allotetraploids: evidence from phosphoenolpyruvate carboxylase gene sequences. Systematic Botany 29: 850–861.
8. Mason-Gamer RJ (2005) The β-amylase genes of grasses and a phylogenetic analysis of the Triticeae (Poaceae). American Journal of Botany 92: 1045–1058.
9. Mason-Gamer RJ (2007) Allopolyploids of the genus *Elymus* (Triticeae, Poaceae): a phylogenetic perspective. Aliso 23: 372–379.
10. Mason-Gamer RJ (2008) Allohexaploidy, introgression, and the complex phylogenetic history on *Elymus repens* (Poaceae). Molecular Phylogenetics and Evolution 47: 598–611.
11. Mason-Gamer RJ, Burns MM, Naum M (2010) Phylogenetic relationships and reticulation among Asian *Elymus* (Poaceae) allotetraploids: analysis of three nuclear genes. Molecular Phylogenetics and Evolution 54: 10–22.
12. Mason-Gamer RJ, Burns MM, Naum M (2010) Reticulate evolutionary history of a complex group of grasses: phylogeny of *Elymus* **StStHH** allotetraploidsbased on three nuclear genes. PLoS ONE 5: e10989.
