# Supplementary material for: Phylogeny of a Genomically Diverse Group of Elymus (Poaceae) Allopolyploids Reveals Multiple Levels of Reticulation
Source: PLoS One. 2013 Nov 1;8(11):e78449. doi: 10.1371/journal.pone.0078449 (PMC3840256; doi:10.1371/journal.pone.0078449)
Supplement: Table S2 — Sequences used in GBSSI exon analysis (Figure 6). (PDF) [file pone.0078449.s007.pdf]

Table S2. Sequences used in analysis of GBSSI exons (Fig. 6).

Taxon	Subfamily, Tribe	GBSSI
Hordeum californicum	Pooideae, Triticeae	AF079273
Psathyrostachys fragilis		AF079279
Pseudoroegneria spicata		AF079281
Taeniatherum caput-medusae		AY011010
Elymus repens 1c		AY360826
Elymus repens 1g		AY360858
Elymus repens 2aa		AY360830
Elymus repens 2h		AY360859
Elymus repens 3a		AY360833
Elymus repens 3g		AY360860
Elymus repens 5b		AY360861
Elymus repens 6dd		AY360845
Bromus catharticus	Pooideae, Bromeae	DQ157055
Bromus tectorum		AY362757
Briza scabra	Pooideae, Poeae	EU395864
Briza erecta		EU395852
Cutandia memphitica		AY362758
Erianthecium bulbosum		EU395853
Glyceria grandis	Pooideae, Meliceae	AF079291
Melica cupanii		AF079296
Arundinaria gigantea	Bambusoideae, Arundinarieae	JN131985
Fargesia yulongshanensis		AF445184
Yushania polytricha		AF445190
Bambusa ventricosa	Bambusoideae, Bambuseae	JN131925
Chusquea exasperata		AF079293
Chusquea oxylepis		AF079294
Pariana radiciflora	Bambusoideae, Olyreae	AF079297

Sequence sources: AF0792xx [1]; AY011010 [2]; AY3608xx and AY362758 [3]; DQ157055 [Liu et al., unpubl.]; EU3958xx [4]; JN1319xx [5]; AF4451xx [6].

1. Mason-Gamer RJ, Weil CF, Kellogg EA (1998) Granule-bound starch synthase: structure, function, and phylogenetic utility. Molecular Biology and Evolution 15: 1658–1673.
2. Mason-Gamer RJ (2001) Origin of North American species of *Elymus* (Poaceae: Triticeae) allotetraploids based on granule-bound starch synthase gene sequences. Systematic Botany 26: 757–768.
3. Mason-Gamer RJ (2004) Reticulate evolution, introgression, and intertribal gene capture in an allohexaploid grass. Systematic Biology 53: 25–37.
4. Essi L, Longhi-Wagner HM, de Souza-Chies TT (2008) Phylogenetic analysis of the *Briza* complex (Poaceae). Molecular Phylogenetics and Evolution 47: 1018–1029.
5. Zhang YX, Zeng CX, Li DZ (2012) Complex evolution in Arundinarieae (Poaceae: Bambusoideae): incongruence between plastid and nuclear GBSSI gene phylogenies. Molecular Phylogenetics and Evolution 63: 777–797.
6. Guo Z-H, Li D-Z (2004) Phylogenetics of the *Thamnocalamus* group and its allies (Gramineae: Bambusoideae): inference from the sequences of GBSSI gene and ITS spacer. Molecular Phylogenetics and Evolution 30: 1–12.
