# Supplementary material for: Phylogeny of a Genomically Diverse Group of Elymus (Poaceae) Allopolyploids Reveals Multiple Levels of Reticulation
Source: PLoS One. 2013 Nov 1;8(11):e78449. doi: 10.1371/journal.pone.0078449 (PMC3840256; doi:10.1371/journal.pone.0078449)
Supplement: Table S3 — Datasets, partitions where applicable, models selected under AIC criterion, and parameters estimated on the best ML tree. (PDF) [file pone.0078449.s008.pdf]

Table S3. Datasets, partitions where applicable, models selected under AIC criterion, and parameters estimated on the best ML tree.

Data Set	Partition	Aligned Length	Model (AIC)	Invariant (I)	Shape (α)	Base frequencies (A, C, G, T)	Rate Matrix
cpDNA	trnT-L-F	2163	GTR+G	0.0 (fixed)	0.1962	0.3665, 0.1347, 0.1596, 0.3391	AC=1.063, AG=0.722, AT=0.440, CG=0.546, CT=0.794, GT=1.000
	rpoA	1375	GTR+G	0.0 (fixed)	0.0500	0.3576, 0.1578, 0.1805, 0.3041	AC=1.568, AG=2.219, AT=0.469, CG=1.295, CT=4.065, GT=1.000
β-Amylase	Exons	608	K80+G	0.0 (fixed)	0.4921	Equal (fixed)	ti/tv=2.7048
	Introns	936	GTR+I+G	0.0 (est.)	1.2874	0.3306, 0.2033, 0.1925, 0.2736	AC=1.307, AG=3.529, AT=0.993, CG=2.193, CT=4.594, GT=1.000
pepC	Single	1348	HK+G	0.0 (fixed)	1.0202	0.2307, 0.2211, 0.2363, 0.3120	ti/tv=1.8002
GBSSI-Triticeae	Exons	759	GTR+G	0.0 (fixed)	0.3759	0.2307, 0.3074, 0.3089, 0.1531	AC=0.619, AG=3.290, AT=0.620, CG=1.063, CT=6.661, GT=1.000
	Introns	507	GTR+G	0.0 (fixed)	0.9455	0.2604, 0.2194, 0.2379, 0.2823	AC=1.226, AG=2.735, AT=0.642, CG=1.350, CT=3.323, GT=1.000
GBSSI exons-Pooideae and Bambusoideae	Single	719	GTR+I+G	0.3077	0.7833	0.2208, 0.2929, 0.3290, 0.1574	AC=1.172, AG=2.858, AT=0.730, CG=1.245, CT=4.357, GT=1.000
